# ACC/AHA/ASE/ASNC/ASPC/HFSA/HRS/SCAI/SCCT/SCMR/STS 2023 Multimodality Appropriate Use Criteria for the Detection and Risk Assessment of Chronic Coronary Disease

**DOI:** 10.1186/s12968-023-00958-5

**Published:** 2023-10-19

**Authors:** David E. Winchester, David J. Maron, Ron Blankstein, Ian C. Chang, Ajay J. Kirtane, Raymond Y. Kwong, Patricia A. Pellikka, Jordan M. Prutkin, Raymond Russell, Alexander T. Sandhu

**Affiliations:** 1 American College of Cardiology, Washington, USA; 2 Society of Cardiovascular Computed Tomography, Washington, USA; 3 Fellow-in-Training, Washington, USA; 4 Society for Cardiovascular Angiography and Interventions, Washington, USA; 5 Society for Cardiovascular Magnetic Resonance, Washington, USA; 6 American Society of Echocardiography, Washington, USA; 7 Heart Rhythm Society, Washington, USA; 8 American Society of Nuclear Cardiology, Washington, USA

**Keywords:** Appropriate Use Criteria, CCD, Chronic coronary disease, Multimodality

## Abstract

The American College of Cardiology (ACC) Foundation, along with key specialty and subspecialty societies, conducted an appropriate use review of stress testing and anatomic diagnostic procedures for risk assessment and evaluation of known or suspected chronic coronary disease (CCD), formerly referred to as stable ischemic heart disease (SIHD). This document reflects an updating of the prior Appropriate Use Criteria (AUC) published for radionuclide imaging, stress echocardiography (echo), calcium scoring, coronary computed tomography angiography (CCTA), stress cardiac magnetic resonance (CMR), and invasive coronary angiography for SIHD. This is in keeping with the commitment to revise and refine the AUC on a frequent basis. As with the prior version of this document, rating of test modalities is provided side-by-side for a given clinical scenario. These ratings are explicitly not considered competitive rankings due to the limited availability of comparative evidence, patient variability, and the range of capabilities available in any given local setting^1–4^.

This version of the AUC for CCD is a focused update of the prior version of the AUC for SIHD^4^. Key changes beyond the updated ratings based on new evidence include the following:

1. Clinical scenarios related to preoperative testing were removed and will be incorporated into another AUC document under development.
2. Some clinical scenarios and tables were removed in an effort to simplify the selection of clinical scenarios. Additionally, the flowchart of tables has been reorganized, and all clinical scenario tables can now be reached by answering a limited number of clinical questions about the patient, starting with the patient’s symptom status.
3. Several clinical scenarios have been revised to incorporate changes in other documents such as pretest probability assessment, atherosclerotic cardiovascular disease (ASCVD) risk assessment, syncope, and others. ASCVD risk factors that are not accounted for in contemporary risk calculators have been added as modifiers to certain clinical scenarios.

The 64 clinical scenarios rated in this document are limited to the detection and risk assessment of CCD and were drawn from common applications or anticipated uses, as well as from current clinical practice guidelines.^5^ These clinical scenarios do not specifically address patients having acute chest pain episodes. They may, however, be applicable in the inpatient setting if the patient is not having an acute coronary syndrome and warrants evaluation for CCD.

Using standardized methodology, clinical scenarios were developed to describe common patient encounters in clinical practice focused on common applications and anticipated uses of testing for CCD. Where appropriate, the scenarios were developed on the basis of the most current ACC/American Heart Association guidelines. A separate, independent rating panel scored the clinical scenarios in this document on a scale of 1 to 9, following a modified Delphi process consistent with the recently updated AUC development methodology. Scores of 7 to 9 indicate that a modality is considered appropriate for the clinical scenario presented, midrange scores of 4 to 6 indicate that a modality may be appropriate for the clinical scenario, and scores of 1 to 3 indicate that a modality is rarely appropriate.

**Supplementary Information:**

The online version contains supplementary material available at 10.1186/s12968-023-00958-5.

**Multimodality Writing Group for Chronic Coronary Disease**

A Report of the American College of Cardiology Solution Set Oversight Committee, American Heart Association, American Society of Echocardiography, American Society of Nuclear Cardiology, American Society of Preventive Cardiology, Heart Failure Society of America, Heart Rhythm Society, Society for Cardiovascular Angiography and Interventions, Society of Cardiovascular Computed Tomography, Society for Cardiovascular Magnetic Resonance, and Society of Thoracic Surgeons.

This document was approved by the American College of Cardiology Clinical Policy Approval Committee in March 2023.

The American College of Cardiology requests that this document be cited as follows: Winchester DE, Maron DJ, Blankstein R, Chang IC, Kirtane AJ, Kwong RY, Pellikka PA, Prutkin JM, Russell R, Sandhu AT. ACC/AHA/ASE/ASNC/ASPC/HFSA/HRS/SCAI/SCCT/SCMR/STS 2023 multimodality appropriate use criteria for the detection and risk assessment of chronic coronary disease: a report of the American College of Cardiology Solution Set Oversight Committee, American Heart Association, American Society of Echocardiography, American Society of Nuclear Cardiology, American Society of Preventive Cardiology, Heart Failure Society of America, Heart Rhythm Society, Society for Cardiovascular Angiography and Interventions, Society of Cardiovascular Computed Tomography, Society for Cardiovascular Magnetic Resonance, and Society of Thoracic Surgeons. *J Am Coll Cardiol*. 2023;81(25):2445–2467.

Copies: This document is available on the website of the American College of Cardiology (www.acc.org). For copies of this document, please contact Elsevier Inc. Reprint Department via fax (212-633-3820) or e-mail (reprints@elsevier.com).

Permissions: Multiple copies, modification, alteration, enhancement, and/or distribution of this document are not permitted without the express permission of the American College of Cardiology. Requests may be completed online via the Elsevier site (https://www.elsevier.com/about/ourbusiness/policies/copyright/permissions).

**Rating Panel**

- L. Samuel Wann, MD, MACC, Moderator*
- Ian C. Chang, MD, FACC, *Writing Group Representative*^‡^
- Alexander T. Sandhu, MD, MS, *Writing Group Representative*^‡^
- W. Patricia Bandettini, MD^‖^
- Dennis A. Calnon, MD, FACC**
- Manuel D. Cerqueira, MD, FACC*
- Larry S. Dean, MD, FACC*
- Milind Y. Desai, MBBS, FACC*
- Howard J. Eisen, MD, FACC*
- Stephen E. Fremes, MD, FACC*
- Mario F.L. Gaudino, MD^††^
- Linda D. Gillam, MD, MACC^¶^
- Nicole L. Lohr, MD, PhD, FACC^‡‡^
- Joseph E. Marine, MD, MBA, FACC^#^
- Khurram Nasir, MBBS, FACC^§§^
- Leslee J. Shaw, PhD, FACC^†^
- Jacqueline E. Tamis-Holland, MD, FACC^§^
- John B. Wong, MD^#††‡‡§§^

^††^Society of Thoracic Surgeons Representative.

^‡‡^American Heart Association Representative.

^§§^American Society of Preventive Cardiology Representative.

**Solution Set Oversight Committee**

- Nicole M. Bhave, MD, FACC, *Chair*
- Niti R. Aggarwal, MD, FACC
- Katie Bates, ARNP, DNP
- Biykem Bozkurt, MD, PhD, FACC
- John P. Erwin iii, MD, FACC
- Chayakrit Krittanawong, MD^‖‖^
- Dharam J. Kumbhani, MD, SM, FACC
- Gurusher S. Panjrath, MBBS, FACC
- Javier A. Sala-Mercado, MD, PhD^‖‖^
- Barbara Wiggins, PharmD, FACC
- David E. Winchester, MD, MS, FACC
- Megan Coylewright, MD, MPH, FACC, *Ex Officio*^‖‖^

^‖‖^Former member of the Solution Set Oversight Committee during development of the document.

**Table of Contents**

- Abstract
- Preface

1. Introduction
2. Methods
  2.1 Clinical Scenario Construction
  2.2 Rating Process and Scoring
3. Assumptions
  Figure 1 Flowchart of Appropriateness Tables
  Table A Advantages and Limitations of Imaging Modalities
4. Definitions
  Table B Examples of Inconclusive Stress Imaging
  Table C Risk-Enhancing Factors
5. Abbreviations
6. Results of Ratings
7. Multimodality for the Detection and Risk Assessment of Ischemic Heart Disease AUC (by Clinical Scenario)
  Section 1
    Table 1.1 Symptomatic Patients With No Known CCD and No Prior Testing
    Table 1.2 Symptomatic Patients Without Known CCD and With Prior Testing
    Table 1.3 Symptomatic Patients With Prior MI or Revascularization
  Section 2
    Table 2.1 Asymptomatic Patients Without Known ASCVD
    Table 2.2 Asymptomatic Patients With Prior Revascularization or MI
    Table 2.3 Asymptomatic Patients Undergoing Assessment of an Exercise Program or Cardiac Rehabilitation
    Table 2.4 Other Cardiovascular Conditions in Patients Without Symptoms of Ischemia
8. Discussion
9. Conclusions

- References
- Appendix
  - Author Relationships with Industry (RWI) and Other Entities (Relevant)

**Preface**

The ACC has a long history of developing documents (eg, decision pathways, health policy statements, AUC) to provide members with guidance on both clinical and nonclinical topics relevant to cardiovascular care. In most circumstances, these documents have been created to complement clinical practice guidelines and to inform clinicians about areas where evidence is new and evolving or where sufficient data is more limited. Despite this, numerous gaps persist, highlighting the need for more streamlined and efficient processes to implement best practices in patient care.

Central to the ACC’s strategic plan is the generation of *actionable knowledge*—a concept that places emphasis on making clinical information easier to consume, share, integrate, and update. To this end, the ACC has shifted from developing isolated documents to creating integrated “solution sets.” These are groups of closely related activities, policy, mobile applications, decision-support tools, and other resources necessary to transform care and/or improve heart health. Solution sets address key questions facing care teams and attempt to provide practical guidance to be applied at the point of care. They use both established and emerging methods to disseminate information for cardiovascular conditions and their related management. The success of solution sets rests firmly on their ability to have a measurable impact on the delivery of care. Because solution sets reflect current evidence and ongoing gaps in care, the associated tools will be refined over time to match changing evidence and member needs.

AUC represent a key component of solution sets. They consist of common clinical scenarios associated with given disease states and ratings that define when it is reasonable to perform testing and, importantly, when it is not. AUC methodology relies on content development work groups, which create patient scenarios, and independent rating panels, which use a modified Delphi process to rate the relevant options for testing and intervention as Appropriate, May Be Appropriate, or Rarely Appropriate. AUC should not replace clinician judgment and practice experience, but should function as tools to improve patient care and health outcomes in a cost-effective manner.

*Nicole Bhave, MD, FACC*

*Chair, ACC Solution Set Oversight Committee*

## 1. Introduction

Since the introduction of AUC in 2005, the ACC has produced a number of documents that synthesize evidence for specific cardiovascular procedures into appropriate use standards. The AUC were developed to support utilization of high-quality patterns of procedure use (ie, appropriate use) while informing efforts to reduce resource use when benefits to patients are unlikely [1–3]. The range of tools used to evaluate cardiovascular disease has expanded over the past decade, especially in the field of noninvasive imaging. The purpose of this document is to delineate the appropriate use of various invasive and noninvasive testing modalities for the diagnosis and/or evaluation of CCD across common patient presentations (clinical scenarios), including the following:

1. Patients with symptoms of ischemia: without prior testing (Table 1.1), with prior testing but without myocardial infarction (MI) or revascularization (Table 1.2), and with prior MI or revascularization (Table 1.3)
2. Patients without symptoms of ischemia: testing for risk of ASCVD events (Table 2.1), and with prior MI or prior revascularization (Table 2.2)
3. Patients seeking to initiate a physical exercise or cardiac rehabilitation program (Table 2.3)
4. Patients with other cardiovascular conditions such as heart failure, arrhythmias, or syncope (Table 2.4)

## 2. Methods

**Writing Group**

At the outset of the AUC development process, the Solution Set Oversight Committee (SSOC) appoints 1 to 2 experts to serve as chair, cochairs, or chair/vice-chair of the writing group. The SSOC, in collaboration with the chair(s), then appoints additional members to serve on the multidisciplinary writing group, which usually ranges in size from 5 to 9 members.

The goal of the writing group is to develop patient scenarios that are likely to be encountered in clinical practice and to categorize those scenarios based on symptoms, anatomy, and/or disease state. Patient presentation varies widely, and not all clinical factors will be fully captured in the scenarios. Where possible, the writing group maps the scenarios to relevant guidelines, clinical trials, and other data sources.

Recommendations for writing group members may be solicited from ACC Member Councils as well as relevant professional societies. In accordance with the ACC’s Diversity and Inclusion principles, every effort is made to ensure that the writing group members vary in age, sex, and ethnicity/race. In addition, one or more early-career physicians, fellows-in-training, or cardiovascular team members are included. Other important considerations for the group’s makeup include specialty, appropriate organizational/content expertise, practice setting, and geographic location. SSOC considers relevant relationships in consideration of ACC’s RWI Policy in the formation of all writing groups.

**Reviewers**

SSOC identifies a group of reviewers to provide feedback to the writing group prior to sending the scenarios to the rating panel. Similar to both the writing group and rating panel, reviewers are solicited from varied sources both internal to the College as well as other relevant societies and organizations. Specifically, reviewers provide feedback on whether the scenarios are comprehensive and represent typical patients, and whether the document provides accurate definitions and assumptions, as well as acceptable evidence mapping.

**Rating Panel**

The rating panel is responsible for rating each clinical scenario. To maximize the input from a broad array of stakeholders, the rating panel is composed of experts in cardiovascular medicine, general internal medicine/hospital practice, and outcomes research. The SSOC is also responsible for appointing members to the rating panel. The membership usually includes 15 to 17 individuals, including practicing clinicians with expertise in the clinical topic being evaluated, practicing clinicians with expertise in a closely related discipline, and often a primary care physician, an expert in statistical analysis, and an expert in clinical trial design. An individual from the public sector and/or a payer representative may also be included.

The panel includes clinicians other than cardiologists to reduce the potential for bias among clinicians with expertise in individual testing modalities or treatment methods. The SSOC has a strong interest in maintaining balance between specialists who use the technology or treatment methods addressed in the specific set of AUC, and other professionals who represent referring clinicians, including general cardiologists, outcome specialists, and/or primary care physicians. Specialists whose key area of practice is the main AUC topic under consideration represent < 50% of the panel.

Similar to the writing group, recommendations for rating panel members are solicited from varied sources. Every effort is made to adhere to the ACC’s Diversity and Inclusion principles, and relevant RWI is taken into consideration. Additionally, SSOC strives to include one or more early career physicians, fellows-in-training, or cardiovascular team members as part of the panel. All rating panels have an odd number of individuals to ensure that the final median score reflects a whole number.

The methods for development of AUC have evolved over time and were recently updated [1–3].

This document summarizes the diagnostic and prognostic capabilities of a multitude of cardiovascular tests to inform choices for testing in common clinical scenarios for the evaluation and management of CCD. Both symptomatic and asymptomatic clinical scenarios are considered, as well as presentations for patients with and without a prior history of CCD. This document intends to provide testing recommendations based on the decisions that would be applicable to providing real-world patient care and should stand as a reference for cardiovascular specialists and referring physicians. The document is intended not to determine a single best test for each clinical scenario, but rather to provide recommendations for a range of testing options that may or may not be reasonable for a specific clinical scenario. It is critical to understand that the AUC should be used to assess an overall pattern of clinical care rather than being the final arbitrator of specific individual cases and should not be used as the sole determination of payment by payors. The ACC and its collaborators believe that an ongoing review of one’s practice using these criteria will help guide more effective testing and, ultimately, better patient outcomes.

### 2.1. Clinical Scenario Construction

The clinical scenarios have been developed by a diverse writing group composed of individuals who are experts in both general cardiology and also noninvasive or invasive cardiac diagnostic testing. The writing group sought to create sets of clinical scenarios that cover the majority of situations for which known or suspected CCD patients are referred for cardiovascular testing. Wherever possible during the writing process, the group members mapped the scenarios to relevant clinical guidelines and key publications or references (see Additional file 1). This included diagnosis-oriented guidelines and modality-specific guidelines. Major consideration was given to trying cover as many clinical scenarios as possible, in balance with usability and ease of navigation of the document. The writing group recognizes that patient presentations vary widely, and not all clinical factors are fully captured by these clinical scenarios.

### 2.2. Rating Process and Scoring

After the scenarios were created, they were reviewed and critiqued by the SSOC and by external reviewers, including general cardiologists, preventive cardiologists, imaging experts, electrophysiologists, cardiac surgeons, and physicians in internal medicine and hospital medicine. After revision by the writing group based on feedback from the reviewers, the scenarios were sent to an independent rating panel [1–3].

To maximize the input from a broad array of stakeholders, the rating panel was comprised of experts in cardiovascular medicine, general medical practice (internal medicine/hospital medicine), and outcomes research. Noncardiologists were included in the process to reduce the potential for bias among physicians with expertise in individual testing modalities. The rating panel was provided with relevant evidence and guidelines to inform their ratings. Formal leadership roles were established for facilitating panel interaction at the subsequent face-to-face meeting. Although panel members were not provided explicit safety and cost information to help determine their appropriate use ratings, they were asked to implicitly consider safety and cost as additional factors in their evaluation of appropriate use. In rating these scenarios, the AUC Rating Panel was asked to assess whether the use of the test for each scenario was Appropriate (A), May Be Appropriate (M), or Rarely Appropriate (R) (see definitions in the following text). When scoring each scenario, the raters were instructed to assume that each modality is locally available, performed on appropriate equipment, and interpreted by individuals with relevant training and expertise.

The first step in the process was for members of the rating panel to evaluate and score the clinical scenarios independently (referred to as the first-round rating). Then, the panel held a virtual, online meeting where panel members were given their scores and a blinded summary of their peers’ scores. The panel discussed the scenarios and the scores, and then panel members were asked again to independently provide scores for each clinical scenario (second-round rating). After the second-round rating, the results were sent back to the writing group for review. At this point, the writing group had a final chance to clarify clinical scenarios and, if necessary, return to the rating panel for rescoring. A more detailed description of the methods is provided in a previous publication, “ACCF Proposed Method for Evaluating the Appropriateness of Cardiovascular Imaging,” which was updated in 2018 [2]. Based on these multiple rounds of review, scoring, and revision, each scenario was classified as Appropriate, May Be Appropriate, or Rarely Appropriate. Although ratings for the clinical scenarios are categorized into 3 groups based on appropriateness, the appropriateness of testing is most accurately viewed as a continuum, depending on the variations of benefits and risks in individual patients.

**Appropriate, median score 7 to 9**: An appropriate option for management of patients in this population because benefits generally outweigh risks; an effective option for individual care plans, although not always necessary, depending on physician judgment and patient-specific preferences (ie, procedure is generally acceptable and generally reasonable for the clinical scenario).

**May Be Appropriate, median score 4 to 6**: At times, an appropriate option for management of patients in this population due to variable evidence or agreement regarding the benefit-risk ratio, potential benefit based on practice experience in the absence of evidence, and/or variability in the population; effectiveness for individual care must be determined by a patient’s physician in consultation with the patient on the basis of additional clinical variables and judgment along with patient preferences (ie, procedure may be acceptable and may be reasonable for the clinical scenario).

**Rarely Appropriate, median score 1 to 3**: Rarely an appropriate option for management of patients in this population due to the lack of a clear benefit/risk advantage; rarely an effective option for individual care plans; exceptions should have documentation of the clinical reasons for proceeding with this care option (ie, procedure is not generally acceptable and is not generally reasonable for the clinical scenario).

The level of agreement among panelists as defined by RAND was analyzed on the basis of the RAND/UCLA modified Delphi Panel method rule for a panel of 14 to 17 members [1, 5]. Ratings were considered to be in agreement when fewer than 5 panelists’ ratings fell outside of the 3-point region containing the median score. Disagreement was defined as when 5 or more panelists’ ratings fell in both the Appropriate and the Rarely Appropriate categories. Any clinical scenario having disagreement was categorized as May Be Appropriate regardless of the final median score.

## 3. Assumptions

To limit inconsistencies in interpretation, the following assumptions and considerations should be applied when interpreting the ratings.

**Fig. 1:**
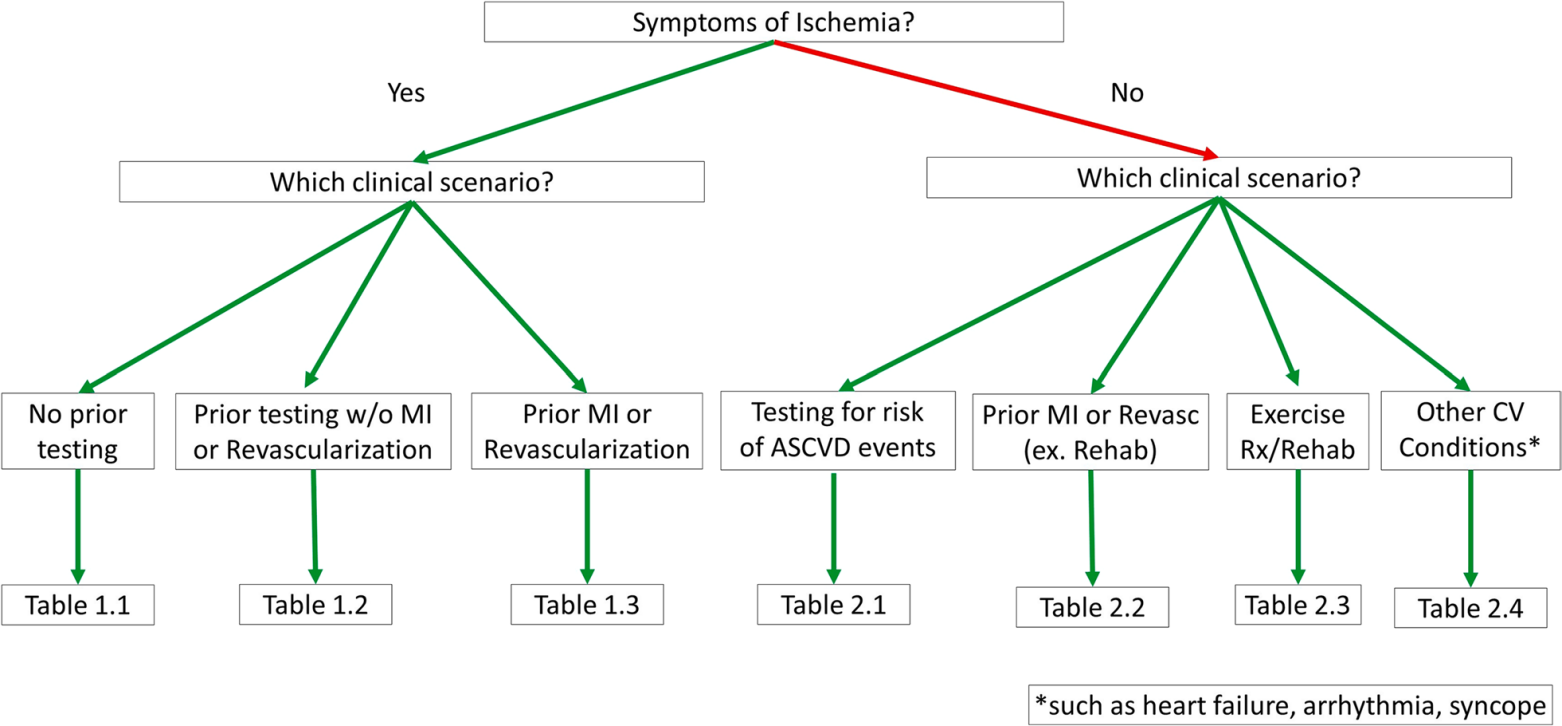
Flowchart of Appropriateness Tables. This flowchart guides users of the document toward the table with clinical scenarios most applicable for the patient in whom imaging of chronic coronary disease (CCD) is being considered. The flowchart prioritizes the presence or absence of symptoms of CCD before further categorization is offered. For those patients who may be classified into more than 1 of the clinical indication tables and/or algorithms, this flowchart places clinical conditions into a hierarchy to aid in assessing appropriateness. ASCVD = atherosclerotic cardiovascular disease; CV = cardiovascular; ex = excluding; MI = myocardial infarction; Rehab = rehabilitation; Revasc = revascularization; Rx = prescription; w/o = without

1. Each test is performed, interpreted, and reported in compliance with published criteria for quality cardiac diagnostic testing, as provided by national laboratory accreditation standards and societal quality guideline documents, including the following.
  1. Exercise ECG [6]
  2. Coronary artery calcium scans [7–9]
  3. Stress echocardiogram [10–12]
  4. Radionuclide myocardial perfusion imaging (MPI) [13–16]
  5. CMR [17–21]
  6. CCTA [22–25]
  7. Invasive coronary angiography [26–28]
  8. Radiation [29–31]
2. Use of these AUC assumes that each modality is locally available, performed on appropriate equipment, and interpreted by individuals with acceptable training and expertise.
3. The diagnostic and prognostic value of a previous test generally decreases over time.
4. The clinical status of the patient should be assumed to be valid as stated in the clinical scenario (eg, a thorough history has been obtained and a physical examination has been conducted such that an asymptomatic patient is truly asymptomatic for the scenario in question).
5. The clinical scenarios in this AUC document are not intended for patients with acute conditions (such as acute coronary syndrome or acute decompensated heart failure), although they may be applicable to evaluating hospitalized patients undergoing an evaluation for CCD.
6. All patients are receiving optimal standard care, including guideline-based risk factor modification for primary or secondary prevention of ischemic heart disease unless specifically noted.
7. In the event of an equivocal or inconclusive noninvasive test (stress electrocardiogram [ECG], stress imaging, or CCTA), where further testing is clinically warranted, a different test modality should be performed.
8. In the event of equivocal or inconclusive results on a coronary angiogram, physiological testing (eg, using fractional flow reserve [FFR] or nonhyperemic indexes, noninvasive stress testing, or intravascular ultrasound for left main coronary artery assessment) may be performed as needed.
9. A variety of additional technologies are available to augment the diagnostic and prognostic information yielded by noninvasive imaging techniques (eg, computed FFR for CCTA, myocardial perfusion for stress echo, novel detector arrangements for single-photon emission computed tomography [SPECT], myocardial blood flow reserve for CMR and position emission tomography [PET], etc.); however, these technologies are not always routinely available. Details about when these technologies are appropriate is beyond the scope of this document, and individual ratings do not assume that these technologies were necessarily used or performed.
10. Before performing a noninvasive stress imaging study, relevant diagnostic information should be reviewed for alternative explanations of the symptoms being evaluated [29]. For example, before stress echo, the baseline resting imaging performed should include a screening assessment of cardiac structure and function, including global and segmental ventricular function, chamber sizes, wall thickness, and cardiac valves, unless assessment of these has already been performed. For CMR and CCTA, scout images should be reviewed for any relevant chest pathology.
11. If the patient’s characteristics are captured under more than 1 clinical scenario, the presence of symptoms should generally be the primary criterion for navigating the flowchart in Fig. 1 and test selection from the tables.
12. Clinical scenarios that describe routine or surveillance imaging imply that the test is being considered solely because a period of time has elapsed, not because of any change in clinical circumstances or any need to consider a change in therapy (Table 2.2).
13. When considering testing that includes an exercise component, it should be assumed that the patient has no limitations that would preclude exercising to a symptomatic endpoint, achieving at least 80% of their age- and sex-predicted workload or ≥ 85% of their age-predicted maximal heart rate. Similarly, unless otherwise stated, it should be assumed that the ECG is interpretable.
14. Selection for and monitoring of contrast agent use is assumed to be in accordance with published standards [20, 25].
15. The clinical scenarios are, at times, purposefully broad to cover an array of cardiovascular signs and symptoms and to account for the ordering physician's best judgment as to the risk of ischemic heart disease. Clear documentation of the reason for ordering the test or procedure should be included in the medical record. Additionally, there are likely clinical scenarios that are not covered in this document.
16. In some clinical scenarios, it may be reasonable to either perform or not perform a test. To reflect this, a column labeled “defer testing” is provided to indicate that testing may be deferred at this time, until a change in the patient’s status warrants reappraisal.
17. Individual test modalities have unique limitations as well as advantages that provide information supplementary to the detection of coronary artery disease and myocardial ischemia. In some cases, these limitations and advantages would make a specific test modality superior to others for an individual patient. Examples are listed in Table A.

**Table A:** Advantages and Limitations of Imaging Modalities

Test Modality	Advantages
Echocardiography	Can evaluate valve disease, diastolic parameters, pulmonary hypertension, myocardial diseases, pericardial disease. Can be performed with pharmacological or exercise stress
SPECT	Can be performed with pharmacological vasodilation or pharmacological/exercise stress
PET	Can quantify peak myocardial blood flow and myocardial blood flow reserve, which improve diagnosis and prognostication and may allow for detection of microvascular disease
CMR	Can assess wall motion, ischemia, and infarction in one study. Can quantify myocardial blood flow to improve test accuracy and assess myocardial and pericardial diseases. Can perform viability testing
CAC	Can detect the presence and amount of calcified coronary plaque; robust prognostic value; does not require a contrast agent
CCTA	Can detect both nonobstructive and obstructive plaque. Can identify noncardiac causes for some symptoms. CT stress perfusion and CT FFR can assess for ischemia
Invasive angiography	Can detect both nonobstructive and obstructive plaque. Can perform physiological testing using FFR or nonhyperemic indices, intravascular imaging (eg, IVUS/OCT), additional testing for coronary spasm and microvascular disease, and adjunctive hemodynamic assessments (eg, right and left heart catheterization)
Test Modality	Limitations
Echocardiography*	Limited acoustic windows (COPD, obesity, breast implants)
SPECT*	Attenuation, motion, and soft tissue artifacts may underestimate extent of disease. Exposure to radiation
PET*	Not widely available with exercise. Exposure to radiation
CMR*	Claustrophobia, artifacts, and safety precautions with metallic medical devices
CCTA	Reduced quality may be present in patients with morbid obesity, high or irregular heart rates, or severe coronary calcification. Exposure to radiation
Invasive angiography	Procedural complications. Exposure to radiation

CCTA = coronary computed tomography angiography; CMR = cardiac magnetic resonance; COPD = chronic obstructive pulmonary disease; CT = computed tomography; FFR = fractional flow reserve; IVUS = intravascular ultrasound; OCT = optical coherence tomography; PET = positron emission tomography; SPECT = single-photon emission computed tomography

*Vasodilator testing is contraindicated if caffeine was used within the last 12 h; stress testing is contraindicated when there is high-risk unstable angina or acute MI (< 2 days)

**Multimodality-Specific Assumptions/Considerations**

**Comparative Rating**

18. Testing modalities are rated for their level of appropriateness specific to clinical scenarios rather than a rank order comparison against other testing modalities. The goal of this document is to identify any and all tests that are considered reasonable for a given clinical scenario. As such, more than 1 test type or even all tests may be considered “Appropriate,” “May Be Appropriate,” or “Rarely Appropriate.”
19. If more than 1 modality falls into the same appropriate use category, it is assumed that clinician judgment; test advantages and disadvantages (Table A); and available local expertise, facilities, and equipment will be considered to determine the optimal test for an individual patient.
20. Clinical scenario ratings contained herein supersede the ratings of similar clinical scenarios contained in previous AUC documents.

**Risk/Benefit**

21. Each test modality considered in this document has inherent risks that may include but are not limited to radiation exposure, sensitivity to iodinated or gadolinium-based contrast agents, other bodily injury, and interpretation error. For any given patient, it is assumed that the ordering and performing clinicians have accounted for these individual risks in their choice of test.
22. Clinical scenarios, such as but not limited to, advanced malignancy, frailty, unwillingness to consider testing, technical reasons rendering testing infeasible, or comorbidities likely to markedly increase procedural risk are beyond the scope of this document but should be taken into consideration in test selection. These may relate to clinical appropriateness for revascularization.
23. Unless explicitly stated, it should be assumed that patients presenting with a specific clinical scenario are potential candidates for all of the test types and do not have any contraindications.

**Radiation Safety**

24. Users of the AUC are aware that the generally applied assumption among experts in radiation biology and epidemiology is a linear no-threshold relationship between radiation exposure and subsequent risk of cancer and that radiation exposure for any given test will be as low as reasonably achievable (ALARA). Tests that impart ionizing radiation will be performed by laboratories that have adopted contemporary dose-reduction techniques [30–33].
25. Testing without radiation or a no-testing strategy should be considered for low-risk premenopausal women [34].

**Cost/Value**

26. In selecting a test, clinical benefits are considered first. Cost and value may also be considered, although estimating these for an individual patient may be difficult due to:
  - Differences in reimbursement depending on region, setting, and payer
  - Differences in cost between cardiovascular testing options
  - Differences in charges versus reimbursement
  - Downstream or serial testing
  - Cost to reduce an adverse event or to add quality-adjusted life expectancy
  - Detection of noncardiac conditions, both positive (occult malignancy) and potentially negative (incidental findings)

**Evidence Review**

27. Clinical scenarios were rated based on the best available data and were mapped to relevant clinical practice guidelines.
28. Newer technologies should not be considered more or less appropriate compared with older technologies.

## 4. Definitions

**Appropriate test:** A test in which the expected clinical benefit exceeds the risks of the procedure by a sufficiently wide margin, such that the procedure is generally considered acceptable or reasonable care. For diagnostic imaging procedures, benefits include incremental information that, when combined with clinical judgment, augments efficient patient care. These benefits are weighed against the potential negative consequences (risks include the potential hazard of missed diagnoses, radiation, contrast agents, and/or unnecessary downstream procedures).

**ASCVD:** Clinical ASCVD is defined by a history of acute coronary syndrome; stable angina; coronary or other arterial revascularization; or stroke, transient ischemic attack, or peripheral arterial disease presumed to be of atherosclerotic origin.

**ASCVD risk estimation**: For decision-making about appropriateness of testing, some clinical scenarios are based on ASCVD risk. Several different risk calculators are available for clinicians to use with individual patients to estimate the long-term likelihood of ASCVD events. Clinicians are suggested to use a calculator that has been validated in the population of patients they are evaluating. For North American populations, the ACC ASCVD Risk Estimator is recommended.

**Clinical scenario**: A specific set of patient characteristics that define a unique situation for which cardiovascular testing may be considered.

**CCD**: Diseases of the heart related to current or prior myocardial ischemia in a stable phase, including history of acute coronary syndrome, obstructive atherosclerosis with or without coronary revascularization, ischemia with no obstructive coronary atherosclerosis, or ischemic heart failure. Patients with CCD may be asymptomatic or may have active symptoms, including angina pectoris, dyspnea, and/or fatigue. These symptoms may or may not be related to exertion.

***Definitions for Table*** 1.1

**Likely anginal symptoms: Chest/epigastric/shoulder/arm/jaw pain, chest pressure/discomfort, when occurring with exertion or emotional stress and relieved by rest, nitroglycerin, or both.**

**Less-likely anginal symptoms**: Symptoms including dyspnea or fatigue when not exertional and not relieved by rest/nitroglycerin; also includes generalized fatigue or chest discomfort occurring in a time course not suggestive of angina (eg, resolves spontaneously within seconds or lasts for an extended period and is unrelated to exertion).

**Noncardiac explanation:** An alternative diagnosis, such as gastroesophageal reflux, chest trauma, anemia, chronic obstructive pulmonary disease, or pleurisy, is present and is the most likely explanation for the patient’s symptoms.

***Definitions for Table*** 1.2

**Coronary artery calcium data and reporting system (CAC-DRS):** A standardized reporting system to report the degree and extent of coronary artery calcification for either quantified measurements (eg, Agatston score) or visual estimates of coronary calcification.

**Coronary artery disease-reporting and data system (CAD-RADS):** A standardized reporting system to provide greater consistency of reporting the degree of coronary stenosis measured on a CCTA.

**Abnormal ECG**: An ECG with findings concerning for ischemia or prior infarction such as resting ST-segment depression or T-wave inversions, Q waves, or left bundle branch block.

**Normal exercise treadmill test**: Adequate exertional effort with no evidence of ischemia and no reproduction of symptoms.

**Inconclusive exercise treadmill test**: An exercise stress test that does not provide a sufficient level of confidence for clinical care, such as < 85% maximum predicted heart rate achieved, ST segments that are uninterpretable due to baseline abnormalities, or ST-segment changes that resolve rapidly or are nonspecific.

**Inconclusive stress imaging:** A SPECT, PET, echo, or CMR imaging stress study that does not provide adequate or reliable information to allow a diagnosis or therapeutic strategies to be established to a sufficiently high level of clinical confidence (Table B).

**Table B:** Examples of Inconclusive Stress Imaging

Test Modality	Inconclusive Result
SPECT/PET	Motion artifact, attenuation defects, arrhythmia, apical thinning artifact
Stress echocardiogram	Poor windows, poor endocardial visualization, failure to achieve adequate heart rate
Stress CMR	Artifacts, arrhythmia

CMR = cardiac magnetic resonance; PET = positron emission tomography; SPECT = single-photon emission computed tomography

**Normal stress imaging**: No evidence of ischemia or infarction.

**Mild ischemia**: Ischemia is present but affects < 10% of the myocardium on stress nuclear imaging, < 4 of 32 subsegments (epicardial and endocardial subsegments of 16 segments) on stress CMR, or < 3 of 16 segments on stress echo or stress CMR.

**Moderate to severe ischemia**: Moderate to severe ischemia has been defined as an estimate of ≥ 5% annual risk of cardiac death or nonfatal MI. This level of risk correlates as follows: for stress nuclear imaging, ≥ 10% ischemic myocardium; for stress echo, ≥ 3 of 16 newly dysfunctional segments during stress; and for stress CMR, ≥ 4 of 32 subsegments with ischemic perfusion defects during vasodilation stress or > 3 of 16 segments with new or worsened dysfunction during exercise stages or progressive inotropic stress.

Categories of invasive coronary angiography results:

1. Mild or none: maximal coronary diameter stenosis is 0% to 39%
2. Intermediate: maximal coronary diameter stenosis is 40% to 69%
3. Obstructive: maximal coronary diameter stenosis is ≥ 70% OR left main coronary artery stenosis ≥ 50%)

**Invasive physiological testing**: The results of coronary physiological testing are generally reported as continuous variables (ranging from 0–1). Although clinical studies of these tests have been performed using dichotomous cutpoints, the results of these tests should not be considered only dichotomously. Lower values correlate with more severe ischemia and worse clinical outcomes, and there may be values above a cutpoint that do not rule out myocardial ischemia. This definition does not assume that a comprehensive assessment for microvascular dysfunction was performed.

***Definitions for Table*** 1.3

**Incomplete revascularization**: Coronary revascularization by percutaneous coronary intervention (PCI) or coronary artery bypass graft with suspected or known residual obstructive epicardial coronary artery stenosis that may or may not be amenable to revascularization, or unrevascularized coronary arteries following an acute coronary syndrome. Examples include an incomplete surgical or percutaneous revascularization (unrevascularized territories due to poor targets, chronic occlusion, or diffuse disease), prior MI without culprit artery revascularization, or prior MI with residual obstructive coronary artery disease (CAD) in a non-infarct-related artery.

**Similar to prior ischemic episode**: Patients who are presenting with symptoms that are similar in character to those which occurred at the time of a prior acute coronary syndrome or stable angina event.

**Likely anginal symptoms**: Chest/epigastric/shoulder/arm/jaw pain, chest pressure/discomfort, when occurring with exertion or emotional stress and relieved by rest, nitroglycerin, or both.

**Less-likely anginal symptoms**: Symptoms including dyspnea or fatigue when not exertional or relieved by rest/nitroglycerin; also includes generalized fatigue or chest discomfort occurring in a time course not suggestive of angina (eg, resolves spontaneously within seconds or lasts for an extended period and is unrelated to exertion).

***Definitions for Table*** 2.1

**ASCVD risk**: See definitions provided in Table 1.2.

**Nontraditional risk factors**: In addition to traditional risk factors, there are several conditions that are associated with premature atherosclerosis or rapid progression of atherosclerosis. In some cases, these risk factors may also be associated with greater morbidity and/or mortality in the setting of an acute coronary syndrome. As such, the presence of such conditions may influence a clinician’s decision to evaluate a patient for the presence of coronary atherosclerosis or SIHD. Examples are provided in Table C.

**Table C:** Risk-Enhancing Factors

Family history of premature ASCVD (men, age < 55 y; women, age < 65 y)
Primary hypercholesterolemia (LDL-C, 160–189 mg/dL [4.1–4.8 mmol/L]); non-HDL-C 190–219 mg/dL [4.9–5.6 mmol/L])
Metabolic syndrome (increased waist circumference, elevated triglycerides [> 175 mg/dL], elevated blood pressure, elevated glucose, and low HDL-C [< 40 mg/dL in men; < 50 mg/dL in women] are factors; tally of 3 makes the diagnosis)
Chronic kidney disease (eGFR 15–59 mL/min/1.73 m^2^ with or without albuminuria; not treated with dialysis or kidney transplantation)
Chronic inflammatory conditions such as psoriasis, RA, lupus, or HIV/AIDS
History of premature menopause (before age 40 y) and history of pregnancy-associated conditions that increase later ASCVD risk such as preeclampsia, gestational diabetes
Noncoronary vascular disease (eg, ABI < 0.9)
High-risk races/ethnicities (eg, South Asian ancestry)
Elevated high-sensitivity C-reactive protein (≥ 2.0 mg/L)
Elevated Lp(a): ≥ 50 mg/dL or ≥ 125 nmol/L
Elevated apoB ≥ 130 mg/dL
Persistently elevated, primary hypertriglyceridemia (≥ 175 mg/dL)
Coronary calcifications on prior imaging (chest x-ray, chest CT)
Prior chest radiation
Chemotherapy with vasotoxicity potential

ABI = ankle-brachial index; apoB = apolipoprotein B; ASCVD = atherosclerotic cardiovascular disease; CT = computed tomography; eGFR = estimated glomerular filtration rate; HDL-C = high-density lipoprotein cholesterol; LDL-C = low-density lipoprotein cholesterol; Lp(a) = lipoprotein a; RA = rheumatoid arthritis

***Definitions for Table*** 2.2

**Incomplete revascularization:** Coronary revascularization by PCI or coronary artery bypass graft with suspected or known residual obstructive epicardial coronary artery stenosis that may or may not be amenable to revascularization, or unrevascularized coronary arteries following an acute coronary syndrome. Examples include an incomplete surgical or percutaneous revascularization (unrevascularized territories due to poor targets, chronic occlusion, or diffuse disease), prior MI without culprit artery revascularization, or prior MI with residual obstructive CAD in a non–infarct-related artery.

**Prior high-risk PCI:** Revascularization posing a higher-than-normal risk for restenosis or closure (eg, PCI of a diffusely diseased saphenous vein graft, treatment of recurrent in-stent restenosis) or a higher risk for adverse sequelae should restenosis occur (eg, left main coronary artery PCI or single remaining vessel/conduit).

***Definitions for Table*** 2.4

**Frequent premature ventricular contractions (PVCs):** More than 30 PVCs per hour [35–37].

**Infrequent PVCs**: Thirty or fewer PVCs per hour.

**Sustained ventricular tachycardia (VT)**: Cardiac arrhythmia of consecutive complexes originating in the ventricles at a rate > 100 beats/min (cycle length: < 600 ms) lasting > 30 s or requiring termination due to hemodynamic compromise in < 30 s.

**Nonsustained VT**: Cardiac arrhythmia of ≥ 3 consecutive complexes originating in the ventricles at a rate > 100 beats/min (cycle length: < 600 ms) that self-terminates in < 30 s and without hemodynamic compromise.

**Heart failure**: Stages B, C, and D heart failure, as defined by the ACCF/AHA Guideline for the Management of Heart Failure [38].

**Syncope:** A symptom that presents with an abrupt, transient, complete loss of consciousness, associated with inability to maintain postural tone, with rapid and spontaneous recovery. The presumed mechanism is cerebral hypoperfusion. There should not be clinical features of other nonsyncopal causes of loss of consciousness, such as seizure, antecedent head trauma, or apparent loss of consciousness (ie, pseudosyncope) [39–41].

## 5. Abbreviations

- AUC = Appropriate Use Criteria
- CAD = coronary artery disease
- CMR = cardiac magnetic resonance
- CCTA = coronary computed tomography angiography
- ECG = electrocardiogram
- Echo = echocardiogram
- MPI = myocardial perfusion imaging
- PCI = percutaneous coronary intervention
- PVC = premature ventricular contraction
- SIHD = stable ischemic heart disease
- VT = ventricular tachycardia

## 6. Results of Ratings

The final ratings for Multimodality AUC on the Detection and Risk Assessment of CCD are listed by clinical scenario in Tables 1.1, 1.2, 1.3, 2.1, 2.2, 2.3, and 2.4. The final score reflects the median score of the 15 rating panel members and has been labeled according to the categories of Appropriate (median 7 to 9), May Be Appropriate (median 4 to 6), and Rarely Appropriate (median 1 to 3) (Additional file 1). The discussion section highlights further general trends in the scoring related to specific patient populations.

## 7. Multimodality for the Detection and Risk Assessment of Ischemic Heart Disease AUC (by Clinical Scenario)

See Tables 1.1, 1.2, 1.3, 2.1, 2.2, 2.3, 2.4.

**Table 1.1:** Symptomatic Patients With No Known CCD and No Prior Testing

Clinical Scenario Text		ECG Treadmill	Stress Nuclear MPI	Stress Echo	Stress CMR	CAC	CCTA	Cath	No Test
1.	Less-likely anginal symptoms with a noncardiac explanation	R (3)	R (2)	R (2)	R (2)	R (3)	R (1)	R (1)	A (8)
2.	Less-likely anginal symptoms, age < 50 y and 0 or 1 CV risk factor	M (4)	R (3)	R (3)	R (3)	M (4)	R (3)	R (1)	A (7)
3.	Less-likely anginal symptoms, age 50 y or above and/or ≥ 2 CV risk factors	M (6)	M (6)	M (6)	M (5)	M (6)	M (5)	R (2)	M (4)
4.	Likely anginal symptoms, age < 50 y and 0 or 1 CV risk factor	A (7)	A (7)	A (7)	A (7)	M (6)	A (7)	R (3)	R (3)
5.	Likely anginal symptoms, age 50 y or above and/or ≥ 2 CV risk factors	A (7)	A (8)	A (8)	A (7)	M (5)	A (7)	A (7)	R (1)

CV risk factors: diabetes mellitus, smoking, family history of premature CAD, hypertension, dyslipidemia

A = Appropriate; CAC = coronary artery calcium; CAD = coronary artery disease; cath = cardiac catheterization; CCD = chronic coronary disease; CCTA = coronary computed tomography angiography; CMR = cardiac magnetic resonance; CV = cardiovascular; ECG = electrocardiogram; echo = echocardiography; M = May Be Appropriate; MPI = myocardial perfusion imaging; R = Rarely Appropriate

**Table 1.2:** Symptomatic Patients Without Known CCD and With Prior Testing*

Clinical Scenario Text		ECG Treadmill	Stress Nuclear MPI	Stress Echo	Stress CMR	CAC	CCTA	Cath	No Test
6.	Abnormal ECG	M (4)	A (8)	A (8)	A (8)	M (5)	A (8)	M (5)	M (4)
7.	Normal ET		M (6)	M (6)	M (6)	M (5)	M (6)	R (3)	M (5)
8.	Inconclusive ET		A (8)	A (8)	A (7)	M (5)	A (8)	M (5)	R (3)
9.	Abnormal ET		A (8)	A (8)	A (7)	M (4)	A (8)	A (8)	M (5)
10.	Normal stress imaging^†^	R (1)	R (2)	R (2)	R (2)	M (4)	A (7)	M (5)	M (6)
11.	Mild ischemia on stress imaging^†^	R (1)	R (3)	R (3)	R (3)	R (3)	A (7)	M (6)	M (5)
12.	Inconclusive stress imaging^†^	R (1)	M (5)	M (5)	M (5)	M (4)	A (8)	M (6)	R (3)
13.	Moderate to severe ischemia on stress imaging^†^	R (1)	R (1)	R (1)	R (1)	R (2)	A (7)	A (9)	M (4)
14.	CCTA with no CAD or up to 49% stenosis (CAD-RADS 0–2)	M (4)	M (5)	M (5)	M (5)	R (1)		R (2)	M (6)
15.	CCTA with moderate stenosis 50%-69% (CAD-RADS 3)	M (6)	A (7)	A (7)	A (7)	R (1)		A (7)	M (5)
16.	CCTA with severe stenosis ≥ 70% (CAD-RADS 4–5)	M (5)	M (6)	M (6)	M (6)	R (1)		A (8)	M (5)
17.	CCTA inconclusive (CAD-RADS N)	A (7)	A (8)	A (8)	A (8)	R (1)		A (7)	R (3)
18.	CAC score = 0 (CAC-DRS 0)	M (5)	M (6)	M (6)	M (6)		M (5)	R (1)	M (5)
19.	CAC score 1–99 (CAC-DRS 1)	M (6)	M (5)	M (6)	M (5)		M (5)	R (3)	M (5)
20.	CAC score 100–299 (CAC-DRS 2)	A (7)	A (7)	A (7)	A (7)		A (7)	M (5)	M (4)
21.	CAC score ≥ 300 (CAC-DRS 3)	A (7)	A (7)	A (7)	A (7)		M (6)	M (6)	R (3)
22.	Invasive coronary angiography with mild or no CAD and/or normal invasive physiological testing^‡^	R (2)	M (3)	R (2)	M (4)	R (1)	R (1)		A (7)
23.	Invasive coronary angiography with intermediate severity and/or invasive physiological testing not done^‡^	M (5)	A (7)	A (8)	A (7)	R (1)	R (1)		M (4)
24.	Invasive coronary angiography with obstructive CAD and/or abnormal invasive physiological testing^‡^	R (2)	M (4)	M (4)	M (4)	R (1)	R (1)		M (4)

If grayed out, rating not applicable

A = Appropriate; ASCVD = atherosclerotic cardiovascular disease; CAC = coronary artery calcium score; CAC-DRS = Coronary Artery Calcium Data and Reporting System; CAD = coronary artery disease; CAD-RADS = Coronary Artery Disease-Reporting and Data System; cath = cardiac catheterization; CCD = chronic coronary disease; CCTA = coronary computed tomography angiography; CMR = cardiac magnetic resonance; CTCA = computed tomography coronary angiography; ECG = electrocardiogram; echo = echocardiography; ET = exercise stress test; M = May Be Appropriate; MPI = myocardial perfusion imaging; PET = positron emission tomography; R = Rarely Appropriate; SPECT = single-photon emission tomography

∗Refers to sequential testing being done as part of a continued patient evaluation or application of recent testing results in the reevaluation of a patient

^†^Stress imaging could be SPECT, PET, echo, or CMR

^‡^Refers to diagnostic angiography, not percutaneous coronary intervention

**Table 1.3:** Symptomatic Patients With Prior MI or Revascularization

Clinical Scenario Text		ECG Treadmill	Stress Nuclear MPI	Stress Echo	Stress CMR	CAC	CCTA	Cath	No Test
25.	Incomplete revascularization	M (4)	A (8)	A (8)	A (7)	R (1)	R (3)	M (6)	M (4)
26.	Prior PCI, symptoms similar to prior ischemic episode and/or anginal symptoms	M (5)	A (8)	A (8)	A (8)	R (1)	M (5)	A (7)	M (5)
27.	Prior PCI, nonanginal symptoms	M (5)	M (6)	M (6)	M (6)	R (1)	M (5)	R (3)	M (6)
28.	Prior CABG, symptoms similar to prior ischemic episode and/or anginal symptoms	M (4)	A (8)	A (8)	A (8)	R (1)	M (6)	A (7)	M (5)
29.	Prior CABG, nonanginal symptoms	M (5)	M (6)	M (6)	M (6)	R (1)	M (6)	R (3)	M (5)
30.	Prior MI, no revascularization, symptoms similar to prior ischemic episode and/or anginal	M (5)	A (8)	A (8)	A (8)	R (1)	A (7)	A (7)	R (3)
31.	Prior MI, no revascularization, nonanginal symptoms	M (5)	M (6)	M (6)	M (6)	R (1)	M (6)	M (5)	M (5)
32.	Assessment of myocardial viability	R (1)	A (8)	A (7)	A (8)	R (1)	R (1)	R (1)	
33.	Prior to cardiac rehabilitation, coronary disease (no new or worsening symptoms)	A (7)	M (5)	M (5)	M (4)	R (1)	R (2)	R (1)	M (4)

If grayed out, rating not applicable

A = Appropriate; CABG = coronary artery bypass graft; CAC = coronary artery calcium score; cath = cardiac catheterization; CCTA = coronary computed tomography angiography; CMR = cardiac magnetic resonance; ECG = electrocardiogram; echo = echocardiography; M = May Be Appropriate; MPI = myocardial perfusion imaging; MI = myocardial infarction; PCI = percutaneous coronary intervention; R = Rarely Appropriate

**Table 2.1:** Asymptomatic Patients Without Known ASCVD

Clinical Scenario Text		ECG Treadmill	Stress Nuclear MPI	Stress Echo	Stress CMR	CAC	CCTA	Cath	No Test
34.	Low ASCVD risk < 5%*	R (2)	R (1)	R (1)	R (1)	M (4)	R (1)	R (1)	A (8)
35.	Borderline ASCVD risk 5% to 7.5%	M (4)	R (2)	R (2)	R (2)	A (7)	R (2)	R (1)	A (7)
36.	Borderline ASCVD risk 5% to 7.5% with risk-enhancing factors^†^	M (4)	R (3)	R (3)	R (3)	A (7)	R (3)	R (1)	A (7)
37.	Intermediate ASCVD risk 7.5% to 20% with or without risk-enhancing factors^†^	M (5)	R (3)	R (3)	R (3)	A (8)	R (3)	R (1)	M (5)
38.	High ASCVD risk > 20%	M (5)	M (4)	M (4)	M (4)	M (6)	M (4)	R (2)	M (5)

A = Appropriate; ASCVD = atherosclerotic cardiovascular disease; CAC = coronary artery calcium score; cath = cardiac catheterization; CCTA = coronary computed tomography angiography; CMR = cardiac magnetic resonance; ECG = electrocardiogram; echo = echocardiography; M = May Be Appropriate; MPI = myocardial perfusion imaging; R = Rarely Appropriate

*Risk calculated using the ASCVD risk estimator

^†^See Table C, Risk-Enhancing Factors

**Table 2.2:** Asymptomatic Patients With Prior Revascularization or MI

Clinical Scenario Text		ECG Treadmill	Stress Nuclear MPI	Stress Echo	Stress CMR	CAC	CCTA	Cath	No Test
39.	Incomplete revascularization	M (5)	M (6)	M (6)	M (6)	R (1)	M (4)	R (2)	M (5)
40.	Prior high-risk PCI	M (4)	M (6)	M (5)	M (5)	R (1)	M (4)	R (3)	M (5)
41.	< 5 y after CABG	R (2)	R (2)	R (2)	R (2)	R (1)	R (3)	R (1)	A (7)
42.	> 5 y after CABG	M (4)	M (4)	M (4)	M (4)	R (1)	M (4)	R (2)	A (7)
43.	< 2 y after PCI	R (2)	R (2)	R (2)	R (2)	R (1)	R (2)	R (1)	A (7)
44.	> 2 y after PCI	M (5)	M (5)	M (5)	M (5)	R (1)	M (4)	R (1)	A (7)
45.	Patients at high risk for or with a history of silent ischemia*	M (4)	A (7)	A (7)	A (7)	R (1)	M (5)	R (3)	M (5)
46.	Assessment of myocardial viability	R (1)	A (7)	M (6)	A (7)	R (1)	R (1)	R (1)	
47.	Isolated evaluation of bypass graft patency	R (3)	M (5)	M (5)	M (5)	R (1)	A (7)	R (3)	M (6)

If grayed out, rating not applicable

A = Appropriate; CABG = coronary artery bypass graft; CAC = coronary artery calcium score; cath = cardiac catheterization; CCTA = coronary computed tomography angiography; CMR = cardiac magnetic resonance; ECG = electrocardiogram; echo = echocardiography; M = May Be Appropriate; MI = myocardial infarction; MPI = myocardial perfusion imaging; PCI = percutaneous coronary intervention; R = Rarely Appropriate

*Diabetes mellitus with accelerated progression of CAD, chronic kidney disease, peripheral artery disease, prior brachytherapy, in-stent restenosis, saphenous vein graft intervention [42]

**Table 2.3:** Asymptomatic Patients Undergoing Assessment of an Exercise Program or Cardiac Rehabilitation

Clinical Scenario Text		Exercise ECG	Stress Nuclear MPI	Stress Echo	Stress CMR	CAC	CCTA	Cath	No Test
48.	Prior to initiation of an unsupervised exercise program, without known CCD	M (6)	R (3)	R (3)	R (3)	R (3)	R (1)	R (1)	A (7)
49.	Prior to initiation of an unsupervised exercise program, with known CCD	A (7)	M (5)	M (5)	M (4)	R (1)	R (2)	R (1)	M (4)
50.	Prior to cardiac rehabilitation	A (7)	M (4)	M (4)	M (4)	R (1)	R (2)	R (1)	M (5)

A = Appropriate; CAC = coronary artery calcium score; cath = cardiac catheterization; CCD = chronic coronary disease; CCTA = coronary computed tomography angiography; CMR = cardiac magnetic resonance; ECG = electrocardiogram; echo = echocardiography; HFpEF = heart failure with preserved ejection fraction; HFrEF = heart failure with reduced ejection fraction; M = May Be Appropriate; MI = myocardial infarction; MPI = myocardial perfusion imaging; R = Rarely Appropriate

**Table 2.4:** Other Cardiovascular Conditions in Patients Without Symptoms of Ischemia

Clinical Scenario Text		ECG Treadmill	Stress Nuclear MPI	Stress Echo	Stress CMR	CAC	CCTA	Cath	No Test
**Newly-Diagnosed Heart Failure (Resting LV Function Previously Assessed but No Prior CAD Evaluation)**									
51.	Newly diagnosed HFpEF	M (4)	A (7)	A (8)	A (7)	R (3)	A (7)	M (6)	R (3)
52.	Newly diagnosed HFrEF	M (4)	A (7)	A (8)	A (8)	R (2)	A (7)	A (8)	R (1)
53.	Screening for transplant vasculopathy	R (3)	A (7)	A (7)	A (7)	R (1)	A (7)	A (8)	
**Evaluation of Arrhythmias Without Ischemic Equivalent (No Prior Cardiac Evaluation)**									
54.	Infrequent PVCs	M (4)	R (2)	R (2)	R (2)	R (2)	R (1)	R (1)	A (8)
55.	Frequent PVCs or nonsustained VT	A (7)	A (7)	A (7)	A (7)	R (3)	M (6)	M (5)	M (4)
56.	Paroxysmal supraventricular tachycardia	M (5)	R (2)	R (3)	R (3)	R (1)	R (2)	R (1)	M (5)
57.	New-onset atrial fibrillation/flutter	M (5)	R (3)	R (3)	R (3)	R (2)	R (3)	R (1)	M (5)
58.	Prior to initiation of antiarrhythmic therapy in patients with high global CAD risk	M (6)	A (7)	A (7)	A (7)	R (3)	A (7)	R (3)	R (3)
59.	Exercise-induced VT	A (7)	A (7)	A (8)	A (7)	R (2)	A (7)	A (7)	R (1)
60.	Sustained VT	A (7)	A (7)	A (7)	A (7)	R (2)	A (7)	A (7)	R (1)
61.	Ventricular fibrillation	M (4)	A (7)	A (7)	A (7)	R (1)	A (7)	A (8)	R (1)
**Syncope Without Ischemic Equivalent**									
62.	Initial evaluation suggests CV abnormalities	A (7)	A (7)	A (7)	A (7)	R (3)	M (6)	M (5)	R (3)
63.	Initial evaluation suggests other etiology	M (4)	R (3)	M (4)	R (3)	R (2)	R (2)	R (1)	M (6)
**Cardio-oncology**									
64.	Prior chest radiation, no symptoms, > 5 y ago	M (4)	M (4)	M (6)	M (5)	M (6)	M (6)	R (2)	M (5)

If grayed out, rating not applicable

A = Appropriate; CAC = coronary artery calcium score; CAD = coronary artery disease; cath = cardiac catheterization; CCTA = coronary computed tomography angiography; CMR = cardiac magnetic resonance; CV = cardiovascular; ECG = electrocardiogram; echo = echocardiography; HFpEF = heart failure with preserved ejection fraction; HFrEF = heart failure with reduced ejection fraction; M = May Be Appropriate; MPI = myocardial perfusion imaging; PVC = premature ventricular contraction; R = Rarely Appropriate; VT = ventricular tachycardia

## 8. Discussion

The foundation for this AUC document is the 2013 AUC for Multimodality Imaging in SIHD, one of the first documents to shift away from a test-modality–specific focus toward a clinical focus [4]. In this revision, the writing group sought to produce a balanced document that offered ease of use and a comprehensive list of clinical scenarios. The writing group established a formal definition of CCD, which had not been done in prior ACC documents, to delineate the scope of the document. Substantial changes were made to the organizational flow chart, and some tables were simplified or removed. In a few instances, the writing group felt that expansion of scenarios was warranted to capture clinically relevant situations that were not acknowledged in the prior version. Because the ACC has a standalone AUC document being developed on the management of heart disease in the perioperative/periprocedural setting, those clinical scenarios were removed from this document. As with the prior version, this document refers only to patients with stable conditions, and a separate AUC addressing acute chest pain syndromes is being considered by the ACC.

Because of these changes, this document consists of 20% fewer clinical scenarios compared with the prior iteration [4]. Although ratings in this document supersede those in the 2013 document, it should be noted that the ACC has sponsored other AUC documents that may have some overlap with scenarios in this document. For example, the 2017 AUC for valvular heart disease provide recommendations on ischemia testing modalities in patients with syncope and palpitations [43]. The American College of Radiology maintains many appropriateness documents that have a categorization structure that differs from the ACC’s [44]. This represents an area of ongoing uncertainty for clinicians and for health policy because similar scenarios in documents developed through different methods may have discordant appropriateness ratings [45].

Aside from changes in clinical scenarios, one of the most substantial changes in this version of the AUC is the inclusion of a “no testing” column alongside the noninvasive and invasive testing columns. In terms of precedent for this change, the 2018 AUC for peripheral artery intervention included “continue or intensify medical therapy” as an option alongside invasive management options [46]. The writing group for the 2013 AUC of multimodality imaging for SIHD acknowledged in the discussion that a “no test at all” rating may also be considered an option for some clinical scenarios [4]. The writing group for this document felt it was time to adopt a “no test” column to formally acknowledge that testing may be safely deferred in some situations. Rating of the “no test” option was omitted for selected scenarios where the writing group did not think it applicable. Clinicians should remain aware that the appropriateness of testing deferral, as with the appropriateness of other testing modalities, may change when there is a change in the patient’s clinical scenario. If such a change occurs, the appropriateness of deferring testing and other options should be evaluated under the newly applicable clinical scenario.

The inclusion of the “no test” column introduces some novel considerations and potential implications. First, there are generally less data examining the clinical impact on outcomes and safety of not performing testing compared with performing testing. Clinical scenarios of patients for whom testing was considered and not pursued is difficult to capture in medical records. This makes evaluation of deferred testing challenging to audit. Second, the presence of a “no test” option provides an opportunity to engage in shared decision-making with patients, allowing personal values and preferences to weigh on the choice to perform a test. Third, the writing group strongly advises against use of this document and its ratings for making blanket insurance coverage or reimbursement decisions. If both testing and “no test” are rated appropriate in a given clinical scenario, clinical decision-making should be informed by the individual patient’s situation.

In this version of the AUC, the summary flowchart (Fig. 1) has been rearranged with a reduced hierarchy to try to more closely follow the flow of clinical decision-making. This was intended to make navigation to the desired clinical scenario easier. The prior version of the AUC for the detection and risk assessment of SIHD noted in the assumptions, “If the patient’s characteristics are captured under more than 1 indication, the patient should be categorized according to the hierarchy provided in Fig. 1” [4]. In the current version, clinicians will have to rely on clinical judgment in situations where a patient fits into more than 1 clinical scenario. By starting the hierarchy with a yes/no question about symptoms, the document potentially favors those clinical scenarios that are more often rated as appropriate (in symptomatic patients) compared with other scenarios in which a patient is asymptomatic. The writing group suggests that when a patient fits more than 1 scenario, the scenario best matching the predominant clinical question should be applied.

Throughout the writing process, the writing group had several discussions about whether to divide certain testing modalities into subtypes. For example, CT could be further identified as coronary CT angiography alone or with CT-based FFR, or nuclear MPI as PET or SPECT. Ultimately, this was not done for several reasons. First, although there are potential clinical reasons to perform 1 type of test over another, those reasons may not always be captured within the clinical scenarios. For example, if PET provides superior image quality to SPECT in patients with obesity, but the clinical scenarios do not specifically address testing in obese vs normal-weight patients, then the appropriateness ratings are not likely to be different and would add unnecessary complexity to the tables. Second, for the clinical scenarios that were included, the writing group did not think that identifying the specific subtypes within a given imaging modality would result in any substantial difference in the ratings (eg, for a patient with recurrent anginal symptoms after PCI, both SPECT and PET could be appropriate). Third, the addition of more columns could increase the complexity and reduce the usability of the tables. Fourth, essentially all modalities have subtypes, and the writing group did not believe it would be appropriate or beneficial to include 1 test modality subtype preferentially without including all subtypes as separate columns. The potentially relevant differences for individual imaging modalities are acknowledged in Table A and should be incorporated with clinical features, clinical judgment, and local availability and expertise when selecting a testing strategy.

As a result of the effort to simplify application of the AUC in this version of the document, the terms for classifying angina were changed. The prior version of this document used the terms *typical angina*, *atypical angina*, and *nonanginal symptoms*, whereas this version of the AUC uses the terms *likely anginal* and *less-likely anginal* symptoms. Although *atypical angina* has a specific definition based on criteria from Diamond and Forrester’s symptom classification, this term is known to be applied incorrectly in clinical practice. For example, for patients with symptoms that may be ischemic, conscious or unconscious bias on the part of the clinician may result in the symptoms being labeled atypical to justify not performing a test. However, for patients with symptoms unlikely to have an ischemic origin, the term atypical angina can be used to justify testing. In Table 1.1, we have included a clinical scenario where a clear, noncardiac etiology is present to demonstrate for clinicians that testing should typically not be performed “just to be sure.” Due to the separate processes and the methodology specific to guideline and AUC development, the terms used in this document do not mirror the “cardiac” and “possibly cardiac” terms used in the 2021 chest pain guideline. For users of this AUC, the writing group considers the terms “likely anginal” and “cardiac” to be equivalent, as well as “less likely anginal” and “possibly cardiac.”

In clinical scenarios for symptomatic patients with no prior testing, the recommendation to calculate the pretest likelihood of obstructive coronary disease has been removed (Table 1.1). The primary reason for this change is that the pretest likelihood strategy, as described in the prior version of the AUC, does not perform well at identifying patients who could safely defer testing or those at high pretest likelihood of obstructive CAD. Contemporary cohort data has demonstrated how changes in the epidemiology of CAD warrant rethinking these traditional strategies [47, 48]. The writing group elected to use the simplified symptom profiles described earlier, recognizing that for many patients with symptoms, testing for CCD is appropriate. By adopting this strategy, this version of the AUC for imaging in CCD is the first to incorporate patient risk factors, not just age and sex, as relevant considerations when deciding on a test for CCD.

The approach to symptomatic patients with prior testing has been redesigned in this AUC document (Table 1.2). Based on the available literature on how AUC for CCD were being used in clinical practice, Tables 2.0 to 2.3 in the 2013 AUC were rarely used. By collapsing these scenarios into a single table, the flowchart was substantially simplified. The 2013 document used a cutoff of 90 days to define sequential tests performed as part of a continued evaluation for a given clinical presentation vs an older test with less clinical relevance. Although this is an important clinical distinction, the writing group believed that the 90-day time cutoff was arbitrary and elected to provide 1 table to cover all recommendations for sequential testing.

Clinical scenarios related to the assessment of patients with prior revascularization have also been revised, now based on symptom status (Table 1.3). Specifically, patients with prior revascularization are now categorized based on whether their symptoms are anginal or similar in quality to prior CCD episodes. This was done with the intent of acknowledging that patients with prior revascularization may experience a wide array of symptoms, some of which are more likely to be ischemic, and some of which are clearly noncardiac in origin. In the former, invasive testing may be warranted, but in the latter, ischemia testing can often be deferred. Acknowledging the results of recent studies, such as the ISCHEMIA (International Study of Comparative Health Effectiveness With Medical and Invasive Approach) trial, either testing or deferral of testing may be suitable for symptomatic patients with prior revascularization based on their preferences and individual clinical situations [49, 50].

The clinical scenarios for asymptomatic patients without known ASCVD (Table 2.1) are significantly modified from the prior document. Instead of using global CAD risk and ECG interpretability or the ability to exercise, these scenarios intended for ASCVD screening have been modified based on the categories of 10-year ASCVD risk and the presence of risk-enhancing factors. Prior chest radiation, coronary artery calcifications on chest imaging, and prior chemotherapy with vasotoxicity potential are included as additional considerations. The reason for these changes was to better align recommendations for CCD testing with the patient groups described in the clinical guidelines on prevention and the management of blood cholesterol [8, 9].

The remainder of the tables, Tables 2.2, 2.3, and 2.4, include a few additional clinical scenarios closing potential gaps in the prior AUC and acknowledging ongoing changes in clinical practice. In Table 2.2, scenarios have been added for assessing graft patency before redo sternotomy, for viability assessment, and for management of patient with or at risk for silent ischemia. Table 2.3 now provides recommendations for unsupervised exercise prescriptions in patients with and without known heart disease. Last, Table 2.4 adds guidance on screening for transplant vasculopathy, testing in new paroxysmal sustained VT and atrial flutter, and a new heading for cardio-oncology and assessment of patients with a history of chest radiation. This table includes scenarios for syncope that have changed to align this AUC document with the 2017 ACC/AHA/HRS syncope guideline, which provides recommendations for cardiovascular testing based on history, physical examination, and ECG [41].

Because of these changes to the clinical scenarios, it is difficult to compare the ratings for individual scenarios and tests with those in prior documents (Table 1.1). Substantial changes to scenarios for the assessment of patients with prior testing and prior MI/revascularization make comparisons to the prior document immaterial (Tables 1.2 and 1.3). Although patients without symptoms in Table 2.1 are categorized in a different fashion than in the 2013 document, the rating panel felt that most testing is not likely warranted for these patients. One exception is CAC scoring, which has greater support across the spectrum of risk. Ratings in Tables 2.2 and 2.3 are largely unchanged. In Table 2.4 of this document, many of the scenario ratings are identical to those from 2013. Testing in the setting of new-onset atrial fibrillation is generally considered rarely appropriate in this document, whereas some test options were previously rated as may be appropriate.

***Future Directions***

The ACC is well into 2 decades of publishing AUC to help guide clinicians on appropriateness of tests and procedures for patients. We anticipate that these documents will continue to play an important role in day-to-day practice and may soon have a larger role in measuring quality at a health system level and through societal clinical registries. Current decision-support systems are often difficult to navigate, and we are hopeful that electronic health record vendors will continue to work on strategies to implement AUC in a way that automatically gathers relevant data for making appropriateness determinations. At present, administrative data lack the clinical granularity necessary to capture the relevant details of clinical scenarios to apply appropriateness criteria. In the future, patient-reported symptom profiles may help enhance the patient voice and further automate the process.

***Limitations***

As with all previous versions of the AUC, there are limitations to the exercise of trying to simplify myriad patient presentations to a brief list of clinical scenarios. Some patients will inevitably not fit the precise definitions provided. The time scale for drafting and revising such documents means the recommendations will inherently lag behind published evidence. For example, work on developing the clinical scenarios and rating the test options preceded the publication of recent chest pain guidelines as well as the pending chronic coronary disease management guidelines by multiple years [51]. Although the writing group worked internally with the ACC to eliminate any disagreements with these documents, they could not be inherently part of the development of these AUC. The ACC is developing new strategies to “chunk” guidelines and other documents so that they will be easier to update on a shorter timetable.

## 9. Conclusions

The 2023 AUC for multimodality imaging in CCD has been substantially revised in an effort to make application easier and more closely aligned to how clinical decisions are made in practice. Special attention has been paid to aligning this document with clinical practice guidelines and contemporary scientific studies. Several innovations have been introduced, most notably a column of ratings for “no test,” reinforcing the concept that not every patient encounter warrants cardiovascular testing.

**ACC President and Staff**

- B. Hadley Wilson, MD, FACC, President
- Cathy C. Gates, Chief Executive Officer
- Joseph M. Allen, MA, Team Lead, Clinical Standards and Solution Sets
- Amy Dearborn, Team Lead, Clinical Policy Content Development
- Marίa Velásquez, Project Manager, Appropriate Use Criteria
- Grace Ronan, Team Lead, Clinical Policy Publications

### Supplementary Information

**Additional file 1. **Guideline Mapping File.

**Additional file 2. **Relationships with Industry and Other Entities (Comprehensive).

## Appendix. Author Relationships With Industry (RWI) and Other Entities (Relevant)—2023 Multimodality Appropriate Use Criteria for the Detection and Risk Assessment of Chronic Coronary Disease

The ACC and the Solution Set Oversight Committee (SSOC) recognize the importance of avoiding real or perceived relationships with industry (RWI) or other entities that may affect clinical policy. The ACC maintains a database that tracks all relevant relationships for ACC members and persons who participate in ACC activities, including those involved in the development of AUC. AUC documents follow ACC RWI Policy in determining what constitutes a relevant relationship, with additional vetting by the SSOC.

An even more specific RWI policy applies to the writing group and rating panel for AUC:

1. AUC writing groups must be chaired or cochaired by an individual with no relevant RWI. Vice chairs, however, may have relevant RWI, along with the other writing group members. While writing group members play an important role in the development and final publication of AUC, they do not have any involvement in the rating process or determination of the final scores.
2. AUC rating panel members are involved in the actual rating of scenarios and as such, < 50% may have relevant RWI. Furthermore, the moderator of the rating panel may not have relevant RWI.

Relevant disclosures for the writing group, rating panel, reviewers, and SSOC members can be found in this Appendix. To ensure complete transparency, a full list of disclosure information, including relationships not pertinent to this document, is available in Additional file 2. Participants are discouraged from acquiring relevant RWI throughout the writing and rating process.

**Table:**

Participant	Employment	Representing	Consultant	Speakers Bureau	Ownership/Partnership/Principal	Personal Research	Institutional, Organizational, or Other Financial Benefit	Expert Witness
**Writing Group**								
David E. Winchester, *Co-Chair*	University of Florida, Division of Cardiology—Professor of Medicine and Radiology	ACC	None	None	None	None	None	None
David J. Maron, *Co-Chair*	Stanford University School of Medicine—Professor of Medicine, Cardiovascular, Director, Preventive Cardiology	ACC	None	None	None	None	None	None
Ron Blankstein	Brigham and Women’s Hospital—Associate Director, Cardiovascular Imaging Program, Professor of Medicine, Harvard Medical School	SCCT	1. Amgen, Inc 2. Caristo Diagnostics 3. Novartis*	None	None	1. Amgen, Inc^†^ 2. Novartis	1. SCCT (Officer)	None
Ian C. Chang	Mayo Clinic—Assistant Professor of Medicine, and Georgetown University—Assistant Professor of Medicine	ACC	None	None	None	None	None	None
Ajay J. Kirtane	Columbia University Medical Center—Professor of Medicine, Chief Academic Office, Director NYP/Columbia Cardiac Cath	SCAI	1. IMDS	None	None	1. Abbott Vascular* 2. Amgen, Inc* 3. Boston Scientific* 4. Cardiovascular Systems, Inc* 5. CSI* 6. Medtronic* 7. Philips/Spectranetics* 8. ReCor Medical*	None	None
Raymond Y. Kwong	Brigham and Women’s Hospital—Director of Cardiac Magnetic Resonance Imaging	SCMR	None	None	None	1. Alnylam, Inc* 2. MyoKardia, Inc*	1. SCMR (Officer)	None
Patricia A. Pellikka	Mayo Clinic College of Medicine—Professor of Medicine	ASE	1. Ultromics 2. UpToDate*	None	None	1. Bracco^†^ 2. Edwards Lifesciences 3. GE Healthcare^†^ 4. Lantheus Medical Imaging^†^ 5. OxThera^†^ 6. Ultromics	1. ASE Foundation^†^ 2. National Heart, Lung, and Blood Institute^†^	None
Jordan M. Prutkin	Washington Medical University—Professor of Medicine	HRS	None	None	None	None	1. UpToDate*	None
Raymond Russell	Alpert Medical School of Brown University—Professor of Medicine	ASNC	None	None	1. Dicerna* 2. Terns Pharmaceutical*	None	1. Dicerna* 2. Terns Pharmaceutical*	None
Alexander T. Sandhu	Stanford University School of Medicine—Instructor of Medicine	ACC	None	None	None	None	None	None
**Rating Panel**								
W. Patricia Bandettini	National Institutes of Health—Medical Officer, Heart Failure & Arrhythmias Branch	SCMR	None	None	None	None	None	None
Dennis A. Calnon	Ohio Health Heart and Vascular Physicians—Riverside Methodist Hospital, Cardiac Imaging, Director	ASNC	None	None	None	None	1. ASNC (Officer)	None
Manuel D. Cerqueira	Cleveland Clinic Foundation—Chairman, Department of Molecular and Functional Imaging	ACC Imaging Council	1. Astellas Pharma*	1. Astellas Pharma*	None	None	None	None
Larry S. Dean	Medicine Regional Heart Center University of Washington School of Medicine—Professor of Medicine and Surgery, Director	ACC	1. Teleflex	None	1. Emageon	1. Edwards Lifesciences*	None	None
Milind Y. Desai	Cleveland Clinic Foundation, Heart and Vascular Institute—Professor of Medicine	ACC	1. Bristol Myers Squibb* 2. Caristo Diagnostics 3. Medtronic	None	None	None	None	None
Howard J. Eisen	Pennsylvania State Heart and Vascular Institute—Medical Director, Advanced Heart Failure, Cardiac Transplant Programs	ACC	None	None	None	None	None	None
Stephen E. Fremes	Sunnybrook Health Sciences Centre, Division of Cardiac and Vascular Surgery—Professor, Department of Surgery	ACC	None	None	None	None	1. Bayer (COMPASS)^‡^ 2. Bayer (Galileo)^‡^ 3. Boston Scientific (NeoAcurate II Study)^‡^ 4. Edwards (The Multidisciplinary, Multimodality but Minimalist [3M] Approach to Transfemoral Transcatheter Aortic Valve Replacement)^‡^ 5. HLT, Inc (Radiant study)^‡^ 6. Medtronic (Medtronic TAVR Low Risk)^‡^ 7. Medtronic (SURTAVI)^‡^ 8. Medtronic (Evolut-R FORWARD)^‡^ 9. Bernard Goldman Chair in Cardiovascular Surgery*	None
Mario F.L. Gaudino	Weill Cornell Medical College—Stephen and Suzanne Weiss Professor in Cardiothoracic Surgery, Professor of Cardiothoracic Surgery	STS	None	None	None	None	None	None
Linda D. Gillam	Morristown Medical Center, Department of Cardiovascular Medicine—Chair	ASE	1. Edwards Lifesciences* 2. Egnite Medtronic* 3. Philips*	None	None	1. Edwards Lifesciences* 2. Medtronic*	1. Circulation Imaging (Officer)	None
Nicole L. Lohr	Medical College of Wisconsin, Cardiovascular Medicine—Professor of Medicine	AHA	None	None	None	None	None	None
Joseph E. Marine	Johns Hopkins University School of Medicine, Cardiovascular Medicine—Professor of Medicine	HRS	None	None	None	None	None	None
Khurram Nasir	Houston Methodist DeBakey Cardiology Associates, Preventive Cardiology—Professor of Cardiology	ASPC	1. Amgen, Inc* 2. Esperion 3. Novartis	1. Amgen, Inc	None	None	None	None
Leslee J. Shaw	Icahn School of Medicine at Mount Sinai Hospital Mount Sinai—Director, Blavatnik Family Women’s Health Research Institute, Professor of Medicine	SCCT	None	None	None	None	None	None
Jacqueline E. Tamis-Holland	Icahn School of Medicine at Mount Sinai Hospital Mount Sinai—Director, Women’s Heart NY, Assistant Professor of Medicine, Director, Interventional Cardiology Fellowship	SCAI	None	None	None	None	None	None
L. Samuel Wann	Cardiovascular Disease Consultant	ACC	None	None	None	None	None	None
John B. Wong	Tufts University School of Medicine—Professor of Medicine	ACC	1. Informed Medical Decisions Foundation: Healthwise 2. Annals of Internal Medicine (American College of Physicians)*	None	None	1. Patient-Centered Outcomes Research Institute (PI)*	1. United States Preventive Services Task Force (Member)	None
**Reviewers**								
Niti R. Aggarwal	Mayo Clinic—Assistant Professor of Medicine	Lead SSOC Reviewer	None	None	None	None	None	None
Daniel S. Berman	Cedars-Sinai Medical Center, Department of Imaging—Director, Cardiac Imaging	ACC Imaging Council & SCCT	1. Cedars Sinai Medical Center—Software Royalties* 2. General Electronic	None	None	None	None	None
Matthew J. Budoff	Los Angeles Biomedical Research Institute—Program Director, Division of Cardiology	SCCT	1. Esperion*	1. Amarin* 2. Amgen, Inc* 3. AstraZeneca Pharmaceuticals* 4. Boehringer Ingelheim Pharmaceuticals* 5. Novo Nordisk*	None	None	None	None
Andrew J. Einstein	Columbia University Irving Medical Center, Department of Medicine—Associate Professor of Medicine in Radiology	ASNC	1. Actinia	None	None	1. Canon Medical Systems* GE Healthcare* Roche Medical Systems* W. L. Gore and Associates* Novo Nordisk*	1. ASNC (Officer)^†^ 2. JACC Imaging (Officer)^†^ 3. Journal of Nuclear Cardiology (Officer)^†^	None
Victor A. Ferrari	Hospital of the University of Pennsylvania—Professor of Medicine; Associate Director, Cardiovascular Imaging	SCMR	None	None	None	None	1. Journal of Cardiovascular Magnetic Resonance (Officer)^†^	None
Theodore J. Kolias	University of Michigan Cardiovascular Center—Associate Professor of Medicine	ASE	None	None	None	None	None	None
Jonathon Leipsic	University of British Columbia, Department of Radiology—Professor of Radiology and Cardiology	SCCT	1. CIRCL* 2. MVRX*	1. GE Healthcare 2. Philips	1. CIRCL CVI* 2. Heartflow*	1. Heartflow Inc*	1. Abbott 2. Boston Scientific 3. Edwards 4. Medtronic	None
Brian Olshansky	University of Iowa Carver College, Division of Electrophysiology—Emeritus Professor of Medicine	HRS	None	None	None	None	1. AstraZeneca (DSMB)	None
Harmony R. Reynolds	NYU Grossman School of Medicine, Department of Medicine—Associate Professor of Medicine	AHA	None	None	None	None	None	None
Peter P. Toth	University of Illinois College of Medicine, Division of Cardiology—Adjunct Professor of Medicine	ASPC	1. Amarin 2. Kowa*	1. Amgen, Inc* 2. Esperion 3. Amgen, Inc*	None	None	None	None
Howard S. Weintraub	NYU Grossman School of Medicine, Department of Medicine—Clinical Professor of Medicine	ASPC	1. Amgen, Inc 2. Novartis	None	None	1. Akcea* 2. Amarin 3. Amgen, Inc*	None	None
David H. Wiener	Jefferson Medical College, Jefferson Heart Institute—Professor of Medicine	ASE	None	None	None	None	None	None
**Solution Set Oversight Committee**								
RWI and disclosure statements for members of the SSOC can be found here: https://www.acc.org/guidelines/about-guidelines-and-clinical-documents/guidelines-and-documents-task-forces								

This table represents *relevant* relationships of participants with industry and other entities that were reported at the time this document was under development. The table does not necessarily reflect relationships with industry at the time of publication

A person has a *relevant* relationship IF: the relationship or interest relates to the same or similar subject matter, intellectual property or asset, topic, or issue addressed in the document; the company/entity (with whom the relationship exists) makes a drug, drug class, or device addressed in the document, or makes a competing drug or device addressed in the document; or the person or a member of the person’s household has a reasonable potential for financial, professional, or other personal gain or loss as a result of the issues/content addressed in the document

A person is deemed to have a *significant* interest in a business if the interest represents ownership of ≥ 5% of the voting stock or share of the business entity, or ownership of ≥ $5000 of the fair market value of the business entity; or if funds received by the person from the business entity exceed 5% of the person’s gross income for the previous year. Relationships in this table with no symbol are considered *modest* (less than significant under the preceding definition). Relationships that exist with *no financial benefit* are also included for the purpose of transparency. Please refer to http://www.acc.org/guidelines/about-guidelines-and-clinical-documents/relationships-with-industry-policy for definitions of disclosure categories or additional information about the ACC Disclosure Policy for Writing Committees

ACC = American College of Cardiology; AHA = American Heart Association; ASE = American Society of Echocardiography; ASNC = American Society of Nuclear Cardiology; ASPC = American Society of Preventive Cardiology; AUC = appropriate use criteria; HRS = Heart Rhythm Society; SCAI = Society for Cardiovascular Angiography and Interventions; SCCT = Society of Cardiovascular Computed Tomography; SCMR = Society for Cardiovascular Magnetic Resonance; SSOC = Solution Set Oversight Committee

*Significant relationship

^†^No financial benefit

^‡^Clinical trial enroller
