# Supplementary material for: ACC/AHA/ASE/ASNC/ASPC/HFSA/HRS/SCAI/SCCT/SCMR/STS 2023 Multimodality Appropriate Use Criteria for the Detection and Risk Assessment of Chronic Coronary Disease
Source: J Cardiovasc Magn Reson. 2023 Oct 19;25:58. doi: 10.1186/s12968-023-00958-5 (PMC10585920; doi:10.1186/s12968-023-00958-5)
Supplement: Supplementary file 1 — Additional file 1. Guideline Mapping File. [file 12968_2023_958_MOESM1_ESM.pdf]

ACC/AHA/ASE/ASNC/ASPC/HFSA/HRS/SCAI/SCCT/SCMR/STS 2023 Multimodality Appropriate Use Criteria for the Detection and Risk Assessment of Chronic Coronary Disease: Guideline Mapping File

Table 1.1 Symptomatic Patients With No Known and No Prior Testing			
**Indication**	**Class of Rec. (COR)**	**Level of Evidence (LEVEL) Review**	**Reference**
1. Less-likely anginal symptoms with a noncardiac explanation			
2. Less-likely anginal symptoms, age <50 y and 0 or 1 CV risk factor			
3. Less-likely anginal symptoms, age 50 y or above and/or ≥ 2 CV risk factors			
4. Likely anginal symptoms, age <50 y and 0 or 1 CV risk factor	**I**	**C**	**2014 ACC/AHA Multi-Societal Focused Update of the Guideline for the Diagnosis and Management of Patients with SIHD. Fihn et al.**<br>**2.3 (pg 1933)**<br>Coronary angiography is useful in patients with presumed SIHD who have unacceptable ischemic symptoms despite GDMT and who are amenable to, and candidates for, coronary revascularization.
5. Likely anginal symptoms, age 50 y or above and/or ≥2 CV risk factors	**I**	**C**	**2014 ACC/AHA Multi-Societal Focused Update of the Guideline for the Diagnosis and Management of Patients with SIHD. Fihn et al.**<br>**2.3 (pg 1933)**<br>Coronary angiography is useful in patients with presumed SIHD who have unacceptable ischemic symptoms despite GDMT and who are amenable to, and candidates for, coronary revascularization.

	IIa	C	**2014 ACC/AHA Multi-Societal Focused Update of the Guideline for the Diagnosis and Management of Patients with SIHD. Fihn et al.**<br>**2.3 (pg 1933)**<br>Coronary angiography is reasonable to define the extent and severity of coronary artery disease (CAD) in patients with suspected SIHD whose clinical characteristics and results of noninvasive testing (exclusive of stress testing) indicate a high likelihood of severe IHD and who are amenable to, and candidates for, coronary revascularization (7–12).

References:

The following recommendations have been reviewed and could be applied to the clinical scenarios listed in Table 1.1 depending on the patient's specific symptom characteristics and clinical suspicion of obstructive CAD.

Lancellotti P, Pellikka PA, et al. The Clinical Use of Stress Echocardiography in Non-Ischaemic Heart Disease: Recommendations from the European Association of Cardiovascular Imaging and the American Society of Echocardiography. J Am Soc Echocardiogr 2017;30:101-38. Stress echo is appropriate in patients presenting with exertional dyspnea or fatigue as diastolic function can be assessed in conjunction with assessment of regional wall motion

Pellikka PA, Nagueh SF, Elhendy AA, Kuehl CA, Sawada SG. American Society of Echocardiography recommendations for performance, interpretation, and application of stress echocardiography. J Am Soc Echocardiogr 2007;20:1021-41.Stress echocardiography is useful for the evaluation of patients with dyspnea of possiblecardiac origin.123 In addition to data on the presence, severity, and extent of myocardial ischemia, LV and left atrial volumes, EF, presence of LV hypertrophy and/or valvular disease, a baseline echocardiogram can identify the presence of pulmonary hypertension or abnormal LV relaxation and elevated filling pressures.

2014 ACC/AHA Multi-Societal Focused Update of the Guideline for the Diagnosis and Management of Patients with SIHD. Fihn et al.
2.2.2.1 (pg e66), class I (1)
Standard exercise ECG testing is recommended for patients with an intermediate pretest probability of IHD who have an interpretable ECG and at least moderate physical functioning or no disabling comorbidity. (Level of Evidence: A)

2014 ACC/AHA Multi-Societal Focused Update of the Guideline for the Diagnosis and Management of Patients with SIHD. Fihn et al.
2.2.2.1 (pg e66), class I (2)
Exercise stress with nuclear MPI or echocardiography is recommended for patients with an intermediate to high pretest probability of IHD who have an uninterpretable ECG and at least moderate physical functioning or no disabling comorbidity. (Level of Evidence: B)

2014 ACC/AHA Multi-Societal Focused Update of the Guideline for the Diagnosis and Management of Patients with SIHD. Fihn et al.
2.2.2.1 (pg e66), class IIa (1):
For patients with a low pretest probability of obstructive IHD who do require testing, standard exercise ECG testing can be useful, provided the patient has an interpretable ECG and at least moderate physical functioning or no disabling comorbidity. (Level of Evidence: C)

2014 ACC/AHA Multi-Societal Focused Update of the Guideline for the Diagnosis and Management of Patients with SIHD. Fihn et al.
2.2.2.1 (pg e66), class IIa (2):

Exercise stress with nuclear MPI or echocardiography is reasonable for patients with an intermediate to high pretest probability of obstructive IHD who have an interpretable ECG and at least moderate physical functioning or no disabling comorbidity. (Level of Evidence: B)

2014 ACC/AHA Multi-Societal Focused Update of the Guideline for the Diagnosis and Management of Patients with SIHD. Fihn et al.
2.2.2.1 (pg e66), class IIa (3):
Pharmacological stress with CMR can be useful for patients with an intermediate to high pretest probability of obstructive IHD who have an uninterpretable ECG and at least moderate physical functioning or no disabling comorbidity. (Level of Evidence: B)

2014 ACC/AHA Multi-Societal Focused Update of the Guideline for the Diagnosis and Management of Patients with SIHD. Fihn et al.
2.2.2.1 (pg e66), class IIb (1):
CCTA might be reasonable for patients with an intermediate pretest probability of IHD who have at least moderate physical functioning or no disabling comorbidity. (Level of Evidence: B)

2014 ACC/AHA Multi-Societal Focused Update of the Guideline for the Diagnosis and Management of Patients with SIHD. Fihn et al.
2.2.2.1 (pg e66), class IIb (2):
For patients with a low pretest probability of obstructive IHD who do require testing, standard exercise stress echocardiography might be reasonable, provided the patient has an interpretable ECG and at least moderate physical functioning or no disabling comorbidity. (Level of Evidence: C)

2014 ACC/AHA Multi-Societal Focused Update of the Guideline for the Diagnosis and Management of Patients with SIHD. Fihn et al.
2.2.2.1 (pg e66), class III (1):
Pharmacological stress with nuclear MPI, echocardiography, or CMR is not recommended for patients who have an interpretable ECG and at least moderate physical functioning or no disabling comorbidity. (Level of Evidence: C)

2014 ACC/AHA Multi-Societal Focused Update of the Guideline for the Diagnosis and Management of Patients with SIHD. Fihn et al.
2.2.2.1 (pg e66), class III (2):
Exercise stress with nuclear MPI is not recommended as an initial test in low-risk patients who have an interpretable ECG and at least moderate physical functioning or no disabling comorbidity. (Level of Evidence: C)

2014 ACC/AHA Multi-Societal Focused Update of the Guideline for the Diagnosis and Management of Patients with SIHD. Fihn et al.
2.2.2.2 (pg e66-7), class I (1)
Pharmacological stress with nuclear MPI or echocardiography is recommended for patients with an intermediate to high pretest probability of IHD who are incapable of at least moderate physical functioning or have disabling comorbidity. (Level of Evidence: B)

2014 ACC/AHA Multi-Societal Focused Update of the Guideline for the Diagnosis and Management of Patients with SIHD. Fihn et al.
2.2.2.2 (pg e67), class IIa (1):
Pharmacological stress echocardiography is reasonable for patients with a low pretest probability of IHD who require testing and are incapable of at least moderate physical functioning or have disabling comorbidity. (Level of Evidence: C)

2014 ACC/AHA Multi-Societal Focused Update of the Guideline for the Diagnosis and Management of Patients with SIHD. Fihn et al.

2.2.2.2 (pg e67), class IIa (2):
CCTA is reasonable for patients with a low to intermediate pretest probability of IHD who are incapable of at least moderate physical functioning or have disabling comorbidity. (Level of Evidence: B)

2014 ACC/AHA Multi-Societal Focused Update of the Guideline for the Diagnosis and Management of Patients with SIHD. Fihn et al.
2.2.2.2 (pg e67), class IIa (3):
Pharmacological stress CMR is reasonable for patients with an intermediate to high pretest probability of IHD who are incapable of at least moderate physical functioning or have disabling comorbidity. (Level of Evidence: B)

2014 ACC/AHA Multi-Societal Focused Update of the Guideline for the Diagnosis and Management of Patients with SIHD. Fihn et al.
2.2.2.2 (pg e67), class III (1):
Standard exercise ECG testing is not recommended for patients who have an uninterpretable ECG or are incapable of at least moderate physical functioning or have disabling comorbidity. (Level of Evidence: C)

2014 ACC/AHA Multi-Societal Focused Update of the Guideline for the Diagnosis and Management of Patients with SIHD. Fihn et al.
2.2.2.3 (pg e67), class IIa (1):
CCTA is reasonable for patients with an intermediate pretest probability of IHD who a) have continued symptoms with prior normal test findings, or b) have inconclusive results from prior exercise or pharmacological stress testing, or c) are unable to undergo stress with nuclear MPI or echocardiography. (Level of Evidence: C)

2014 ACC/AHA Multi-Societal Focused Update of the Guideline for the Diagnosis and Management of Patients with SIHD. Fihn et al.
2.2.2.3 (pg e67), class IIb (1):
For patients with a low to intermediate pretest probability of obstructive IHD, noncontrast cardiac CT to determine the CAC score may be considered. (Level of Evidence: C)

2014 ACC/AHA Multi-Societal Focused Update of the Guideline for the Diagnosis and Management of Patients with SIHD. Fihn et al.
3.3.2 (pg e78), class III (3):
Coronary angiography is not recommended to assess risk in patients who are at low risk according to clinical criteria and who have not undergone noninvasive risk testing. (Level of Evidence: C)

2014 ACC/AHA Multi-Societal Focused Update of the Guideline for the Diagnosis and Management of Patients with SIHD. Fihn et al.
2.3 (pg e1753), class I (1)
Coronary angiography is useful in patients with presumed SIHD who have unacceptable ischemic symptoms despite GDMT and who are amenable to, and candidates for, coronary revascularization. (Level of Evidence: C)

2014 ACC/AHA Multi-Societal Focused Update of the Guideline for the Diagnosis and Management of Patients with SIHD. Fihn et al.
2.3 (pg e1753), class IIa (2)
Coronary angiography is reasonable in patients with suspected symptomatic SIHD who cannot undergo diagnostic stress testing, or have indeterminate or nondiagnostic stress tests, when there is a high likelihood that the findings will result in important changes to therapy. (Level of Evidence: C)

2014 ACC/AHA Multi-Societal Focused Update of the Guideline for the Diagnosis and Management of Patients with SIHD. Fihn et al.

2.3 (pg 1933), Class I
Coronary angiography is useful in patients with presumed SIHD who have unacceptable ischemic symptoms despite GDMT and who are amenable to, and candidates for, coronary revascularization. (Level of Evidence: C)

2014 ACC/AHA Multi-Societal Focused Update of the Guideline for the Diagnosis and Management of Patients with SIHD. Fihn et al.
2.3 (pg 1933), Class IIa
Coronary angiography is reasonable to define the extent and severity of coronary artery disease (CAD) in patients with suspected SIHD whose clinical characteristics and results of noninvasive testing (exclusive of stress testing) indicate a high likelihood of severe IHD and who are amenable to, and candidates for, coronary revascularization (7–12). (Level of Evidence: C)

2014 ACC/AHA Multi-Societal Focused Update of the Guideline for the Diagnosis and Management of Patients with SIHD. Fihn et al.
2.3 (pg 1933), Class IIa
Coronary angiography is reasonable in patients with suspected symptomatic SIHD who cannot undergo diagnostic stress testing, or have indeterminate or nondiagnostic stress tests, when there is a high likelihood that the findings will result in important changes to therapy. (Level of Evidence: C)

**Table 1.2 Symptomatic Patients Without Known CCD and With Prior Testing**			
**Indication**	**Class of Rec (COR)**	**Level of Evidence (LEVEL) Review**	**Reference**
6. Abnormal ECG	**IIb**	**B**	**2013 ACC/AHA Guideline on the Assessment of Cardiovascular Risk. Goff et al.**<br>**(pg 2945)**<br>6.1.2. Recommendations for CQ1: Use of Newer Risk Markers After Quantitative Risk Assessment<br>Recommendation 1. If, after quantitative risk assessment, a risk-based treatment decision is uncertain, assessment of 1 or more of the following-family history, hs-CRP, CAC score, or ABI-may be considered to inform treatment decision making.<br>NHLBI Grade: E (Expert Opinion)

	**I**	**C**	**2014 ACC/AHA Multi-Societal Focused Update of the Guideline for the Diagnosis and Management of Patients with SIHD. Fihn et al.**<br>**2.3 (pg 1933)**<br>Coronary angiography is useful in patients with presumed SIHD who have unacceptable ischemic symptoms despite GDMT and who are amenable to, and candidates for, coronary revascularization.
	**IIa**	**C**	**2014 ACC/AHA Multi-Societal Focused Update of the Guideline for the Diagnosis and Management of Patients with SIHD. Fihn et al.**<br>**2.3 (pg 1933)**<br>Coronary angiography is reasonable to define the extent and severity of coronary artery disease (CAD) in patients with suspected SIHD whose clinical characteristics and results of noninvasive testing (exclusive of stress testing) indicate a high likelihood of severe IHD and who are amenable to, and candidates for, coronary revascularization (7–12).
	**IIa**	**C**	**2014 ACC/AHA Multi-Societal Focused Update of the Guideline for the Diagnosis and Management of Patients with SIHD. Fihn et al.**<br>**2.3 (pg 1933)**<br>Coronary angiography is reasonable in patients with suspected symptomatic SIHD who cannot undergo diagnostic stress testing, or have indeterminate or nondiagnostic stress tests, when there is a high likelihood that the findings will result in important changes to therapy.
	**IIb**	**C**	**2014 ACC/AHA Multi-Societal Focused Update of the Guideline for the Diagnosis and Management of Patients with SIHD. Fihn et al.**<br>**2.3 (pg 1933)**<br>Coronary angiography might be considered in patients with stress test results of acceptable quality that do not suggest the presence of CAD when clinical suspicion of CAD

			remains high and there is a high likelihood that the findings will result in important changes to therapy.
	IIa	C	**2014 ACC/AHA Multi-Societal Focused Update of the Guideline for the Diagnosis and Management of Patients with SIHD. Fihn et al.**<br>**2.2.2.3 (pg e67)**<br>CCTA is reasonable for patients with an intermediate pretest prob- ability of IHD who a) have continued symptoms with prior normal test findings, or b) have inconclusive results from prior exercise or pharmacological stress testing, or c) are unable to undergo stress with nuclear MPI or echocardiography.
	III	B	**2014 ACC/AHA Multi-Societal Focused Update of the Guideline for the Diagnosis and Management of Patients with SIHD. Fihn et al.**<br>**3.3.2 (pg e78)**<br>Coronary angiography is not recommended to further assess risk in patients with SIHD who have preserved LV function (EF [illegible]50%) and low-risk criteria on noninvasive testing.
	IIb	C	**2014 ACC/AHA Multi-Societal Focused Update of the Guideline for the Diagnosis and Management of Patients with SIHD. Fihn et al.**<br>**2.3 (pg e1753)**<br>Coronary angiography might be considered in patients with stress test results of acceptable quality that do not suggest the presence of CAD when clinical suspicion of CAD remains high and there is a high likelihood that the findings will result in important changes to therapy
7. Normal ET	IIa	C	**2014 ACC/AHA Multi-Societal Focused Update of the Guideline for the Diagnosis and Management of Patients with SIHD. Fihn et al.**<br>**2.3 (pg 1933)**<br>Coronary angiography is reasonable in patients with suspected symptomatic SIHD who cannot undergo

	 <br><br>**IIb**	 <br><br>**C**	diagnostic stress testing, or have indeterminate or nondiagnostic stress tests, when there is a high likelihood that the findings will result in important changes to therapy.<br><br>**2014 ACC/AHA Multi-Societal Focused Update of the Guideline for the Diagnosis and Management of Patients with SIHD. Fihn et al.**<br>**2.3 (pg 1933)**<br>Coronary angiography might be considered in patients with stress test results of acceptable quality that do not suggest the presence of CAD when clinical suspicion of CAD remains high and there is a high likelihood that the findings will result in important changes to therapy.
8. Inconclusive ET	**IIa**<br><br>**IIa**<br><br>**IIa**	**C**<br><br>**C**<br><br>**C**	**2014 ACC/AHA Multi-Societal Focused Update of the Guideline for the Diagnosis and Management of Patients with SIHD. Fihn et al.**<br>**2.2.2.3 (pg e67)**<br>CCTA is reasonable for patients with an intermediate pretest prob- ability of IHD who a) have continued symptoms with prior normal test findings, or b) have inconclusive results from prior exercise or pharmacological stress testing, or c) are unable to undergo stress with nuclear MPI or echocardiography.<br><br>**2014 ACC/AHA Multi-Societal Focused Update of the Guideline for the Diagnosis and Management of Patients with SIHD. Fihn et al.**<br>**3.3.2 (pg e78)**<br>Coronary angiography is reasonable to further assess risk in pa- tients with SIHD and inconclusive prognostic information after noninvasive testing or in patients for whom noninvasive testing is contraindicated or inadequate.<br><br>**2014 ACC/AHA Multi-Societal Focused Update of the Guideline for the Diagnosis and Management of Patients with SIHD. Fihn et al.**<br>**2.3 (pg e1753)**<br>Coronary angiography is reasonable in patients with suspected symptomatic SIHD who cannot undergo

	 <br><br><br>I	 <br><br><br>C	diagnostic stress testing, or have indeterminate or nondiagnostic stress tests, when there is a high likelihood that the findings will result in important changes to therapy.<br><br>**Role of noninvasive testing in the clinical evaluation of women with suspected ischemic heart disease: a consensus statement from the American Heart Association. Mieres JH, et al. Circulation. 2014 Jul 22;130(4):350-79**. Epub 2014 Jun 16<br>If an ETT is indeterminate (eg, negative ECG in the setting of submaximal exercise [below age-predicted level or failure to achieve >85% predicted maximal heart rate]) or abnormal, the next step should be additional diagnostic testing with stress imaging. Individualized decision making and targeted anti-ischemic therapies after the ETT should consider the woman's ongoing symptom burden and the degree of abnormalities noted during the ETT.
9. Abnormal ET	**IIb**<br><br><br><br><br><br><br><br><br><br><br>**I**<br><br><br><br><br><br><br><br><br>**IIa**	**C**<br><br><br><br><br><br><br><br><br><br><br>**C**<br><br><br><br><br><br><br><br><br>**C**	**2014 ACC/AHA Multi-Societal Focused Update of the Guideline for the Diagnosis and Management of Patients with SIHD. Fihn et al.**<br>**3.2.2.3 (pg e75)**<br>CCTA might be considered for risk assessment in patients with SIHD unable to undergo stress imaging or as an alternative to invasive coronary angiography when functional testing indicates a moderate- to high-risk result and knowledge of angiographic coronary anatomy is unknown.<br><br>**2014 ACC/AHA Multi-Societal Focused Update of the Guideline for the Diagnosis and Management of Patients with SIHD. Fihn et al.**<br>**3.3.2 (pg e78)**<br>Coronary arteriography is recommended for patients with SIHD whose clinical characteristics and results of noninvasive testing indicate a high likelihood of severe IHD and when the benefits are deemed to exceed risk.

	**I**	**C**	**2014 ACC/AHA Multi-Societal Focused Update of the Guideline for the Diagnosis and Management of Patients with SIHD. Fihn et al.**<br>**3.3.2 (pg e78)**<br>Coronary angiography is reasonable to further assess risk in patients with SIHD who have depressed LV function (EF [illegible]50%) and moderate risk criteria on noninvasive testing with demonstrable ischemia.
	**IIa**	**C**	**2014 ACC/AHA Multi-Societal Focused Update of the Guideline for the Diagnosis and Management of Patients with SIHD. Fihn et al.**<br>**2.3 (pg 1933)**<br>Coronary angiography is useful in patients with presumed SIHD who have unacceptable ischemic symptoms despite GDMT and who are amenable to, and candidates for, coronary revascularization.
	**IIb**	**C**	**2014 ACC/AHA Multi-Societal Focused Update of the Guideline for the Diagnosis and Management of Patients with SIHD. Fihn et al.**<br>**2.3 (pg 1933)**<br>Coronary angiography is reasonable in patients with suspected symptomatic SIHD who cannot undergo diagnostic stress testing, or have indeterminate or nondiagnostic stress tests, when there is a high likelihood that the findings will result in important changes to therapy.
	**I**	**C**	**2014 ACC/AHA Multi-Societal Focused Update of the Guideline for the Diagnosis and Management of Patients with SIHD. Fihn et al.**<br>**2.3 (pg 1933)**<br>Coronary angiography might be considered in patients with stress test results of acceptable quality that do not suggest the presence of CAD when clinical suspicion of CAD remains high and there is a high likelihood that the findings will result in important changes to therapy.

			**Role of noninvasive testing in the clinical evaluation of women with suspected ischemic heart disease: a consensus statement from the American Heart Association. Mieres JH, et al. Circulation. 2014 Jul 22;130(4):350-79**. doi: 10.1161/CIR.0000000000000061. Epub 2014 Jun 16<br>If an ETT is indeterminate (eg, negative ECG in the setting of submaximal exercise [below age-predicted level or failure to achieve >85% predicted maximal heart rate]) or abnormal, the next step should be additional diagnostic testing with stress imaging. Individualized decision making and targeted anti-ischemic therapies after the ETT should consider the woman's ongoing<br>symptom burden and the degree of abnormalities noted during the ETT.
10. Normal stress imaging**	**IIa**<br><br><br><br><br><br><br><br><br><br>**IIb**<br><br><br><br><br><br><br><br><br><br>**I**	**C**<br><br><br><br><br><br><br><br><br><br>**C**<br><br><br><br><br><br><br><br><br><br>**C**	**2014 ACC/AHA Multi-Societal Focused Update of the Guideline for the Diagnosis and Management of Patients with SIHD. Fihn et al.**<br>**2.2.2.3 (pg e67)**<br>CCTA is reasonable for patients with an intermediate pretest prob- ability of IHD who a) have continued symptoms with prior normal test findings, or b) have inconclusive results from prior exercise or pharmacological stress testing, or c) are unable to undergo stress with nuclear MPI or echocardiography.<br><br>**2014 ACC/AHA Multi-Societal Focused Update of the Guideline for the Diagnosis and Management of Patients with SIHD. Fihn et al.**<br>**2.3 (pg e1753)**<br>Coronary angiography might be considered in patients with stress test results of acceptable quality that do not suggest the presence of CAD when clinical suspicion of CAD remains high and there is a high likelihood that the findings will result in important changes to therapy.<br><br>**2014 ACC/AHA Multi-Societal Focused Update of the Guideline for the Diagnosis and Management of Patients with SIHD. Fihn et al.**

			**2.3 (pg 1933)**<br>Coronary angiography is useful in patients with presumed SIHD who have unacceptable ischemic symptoms despite GDMT and who are amenable to, and candidates for, coronary revascularization.
	**IIa**	**C**	**2014 ACC/AHA Multi-Societal Focused Update of the Guideline for the Diagnosis and Management of Patients with SIHD. Fihn et al.**<br>**2.3 (pg 1933)**<br>Coronary angiography is reasonable to define the extent and severity of coronary artery disease (CAD) in patients with suspected SIHD whose clinical characteristics and results of noninvasive testing (exclusive of stress testing) indicate a high likelihood of severe IHD and who are amenable to, and candidates for, coronary revascularization (7–12).
	**IIa**	**C**	**2014 ACC/AHA Multi-Societal Focused Update of the Guideline for the Diagnosis and Management of Patients with SIHD. Fihn et al.**<br>**2.3 (pg 1933)**<br>Coronary angiography is reasonable in patients with suspected symptomatic SIHD who cannot undergo diagnostic stress testing, or have indeterminate or nondiagnostic stress tests, when there is a high likelihood that the findings will result in important changes to therapy.
	**IIb**	**C**	**2014 ACC/AHA Multi-Societal Focused Update of the Guideline for the Diagnosis and Management of Patients with SIHD. Fihn et al.**<br>**2.3 (pg 1933)**<br>Coronary angiography might be considered in patients with stress test results of acceptable quality that do not suggest the presence of CAD when clinical suspicion of CAD remains high and there is a high likelihood that the findings will result in important changes to therapy.

	**III**	**B**	**2014 ACC/AHA Multi-Societal Focused Update of the Guideline for the Diagnosis and Management of Patients with SIHD. Fihn et al.**<br>**3.3.2 (pg e78)**<br>Coronary angiography is not recommended to further assess risk in patients with SIHD who have preserved LV function (EF [illegible]50%) and low-risk criteria on noninvasive testing.
	**I**	**C**	**2014 ACC/AHA Multi-Societal Focused Update of the Guideline for the Diagnosis and Management of Patients with SIHD. Fihn et al.**<br>**2.3 (pg 1933)**<br>Coronary angiography is useful in patients with presumed SIHD who have unacceptable ischemic symptoms despite GDMT and who are amenable to, and candidates for, coronary revascularization.
	**IIa**	**C**	**2014 ACC/AHA Multi-Societal Focused Update of the Guideline for the Diagnosis and Management of Patients with SIHD. Fihn et al.**<br>**2.3 (pg 1933)**<br>Coronary angiography is reasonable to define the extent and severity of coronary artery disease (CAD) in patients with suspected SIHD whose clinical characteristics and results of noninvasive testing (exclusive of stress testing) indicate a high likelihood of severe IHD and who are amenable to, and candidates for, coronary revascularization (7–12).
11. Mild ischemia on stress imaging**	**IIa**	**C**	**2014 ACC/AHA Multi-Societal Focused Update of the Guideline for the Diagnosis and Management of Patients with SIHD. Fihn et al.**<br>**2.3 (pg 1933)**<br>Coronary angiography is reasonable in patients with suspected symptomatic SIHD who cannot undergo diagnostic stress testing, or have indeterminate or nondiagnostic stress tests, when there is a high likelihood that the findings will result in important changes to therapy.

	**IIb**	**C**	**2014 ACC/AHA Multi-Societal Focused Update of the Guideline for the Diagnosis and Management of Patients with SIHD. Fihn et al.**<br>**2.3 (pg 1933)**<br>Coronary angiography might be considered in patients with stress test results of acceptable quality that do not suggest the presence of CAD when clinical suspicion of CAD remains high and there is a high likelihood that the findings will result in important changes to therapy.
12. Inconclusive stress imaging**	**IIa**	**C**	**2014 ACC/AHA Multi-Societal Focused Update of the Guideline for the Diagnosis and Management of Patients with SIHD. Fihn et al.**<br>**2.2.2.3 (pg e67)**<br>CCTA is reasonable for patients with an intermediate pretest prob- ability of IHD who a) have continued symptoms with prior normal test findings, or b) have inconclusive results from prior exercise or pharmacological stress testing, or c) are unable to undergo stress with nuclear MPI or echocardiography.
	**IIa**	**C**	**2014 ACC/AHA Multi-Societal Focused Update of the Guideline for the Diagnosis and Management of Patients with SIHD. Fihn et al.**<br>**3.3.2 (pg e78)**<br>Coronary angiography is reasonable to further assess risk in pa- tients with SIHD and inconclusive prognostic information after noninvasive testing or in patients for whom noninvasive testing is contraindicated or inadequate.
	**IIa**	C	**2014 ACC/AHA Multi-Societal Focused Update of the Guideline for the Diagnosis and Management of Patients with SIHD. Fihn et al.**<br>**2.3 (pg e1753)**<br>Coronary angiography is reasonable in patients with suspected symptomatic SIHD who cannot undergo diagnostic stress testing, or have indeterminate or nondiagnostic stress tests, when there is a high likelihood that the findings will result in important changes to therapy.

	I	C	**2014 ACC/AHA Multi-Societal Focused Update of the Guideline for the Diagnosis and Management of Patients with SIHD. Fihn et al.**<br>**2.3 (pg 1933)**<br>Coronary angiography is useful in patients with presumed SIHD who have unacceptable ischemic symptoms despite GDMT and who are amenable to, and candidates for, coronary revascularization.
	IIa	C	**2014 ACC/AHA Multi-Societal Focused Update of the Guideline for the Diagnosis and Management of Patients with SIHD. Fihn et al.**<br>**2.3 (pg 1933)**<br>Coronary angiography is reasonable to define the extent and severity of coronary artery disease (CAD) in patients with suspected SIHD whose clinical characteristics and results of noninvasive testing (exclusive of stress testing) indicate a high likelihood of severe IHD and who are amenable to, and candidates for, coronary revascularization (7–12).
	IIa	C	**2014 ACC/AHA Multi-Societal Focused Update of the Guideline for the Diagnosis and Management of Patients with SIHD. Fihn et al.**<br>**2.3 (pg 1933)**<br>Coronary angiography is reasonable in patients with suspected symptomatic SIHD who cannot undergo diagnostic stress testing, or have indeterminate or nondiagnostic stress tests, when there is a high likelihood that the findings will result in important changes to therapy.
	IIb	C	**2014 ACC/AHA Multi-Societal Focused Update of the Guideline for the Diagnosis and Management of Patients with SIHD. Fihn et al.**<br>**2.3 (pg 1933)**<br>Coronary angiography might be considered in patients with stress test results of acceptable quality that do not suggest the presence of CAD when clinical suspicion of CAD

			remains high and there is a high likelihood that the findings will result in important changes to therapy.
	**IIb**	**C**	**2014 ACC/AHA Multi-Societal Focused Update of the Guideline for the Diagnosis and Management of Patients with SIHD. Fihn et al.**<br>**3.2.2.3 (pg e75)**<br>CCTA might be considered for risk assessment in patients with SIHD unable to undergo stress imaging or as an alternative to invasive coronary angiography when functional testing indicates a **moderate- to high-risk result** and knowledge of angiographic coronary anatomy is unknown.
	**I**	**C**	**2014 ACC/AHA Multi-Societal Focused Update of the Guideline for the Diagnosis and Management of Patients with SIHD. Fihn et al.**<br>**3.3.2 (pg e78)**<br>Coronary arteriography is recommended for patients with SIHD whose clinical characteristics and results of noninvasive testing indicate a high likelihood of severe IHD and when the benefits are deemed to exceed risk.
	**IIa**	**C**	**3.3.2 (pg e78)**<br>Coronary angiography is reasonable to further assess risk in pa- tients with SIHD who have depressed LV function (EF [illegible]50%) and moderate risk criteria on noninvasive testing with demonstrable ischemia.
13. Moderate to severe ischemia on stress imaging**	**IIa**	C	**2014 ACC/AHA Multi-Societal Focused Update of the Guideline for the Diagnosis and Management of Patients with SIHD. Fihn et al.**<br>**2.3 (pg e1753)**<br>Coronary angiography is reasonable to define the extent and severity of coronary artery disease (CAD) in patients with suspected SIHD whose clinical characteristics and results of noninvasive testing (exclusive of stress testing) indicate a high likelihood of severe IHD and who are amenable to, and candidates for, coronary revascularization.

	**III**	**B**	**2014 ACC/AHA Multi-Societal Focused Update of the Guideline for the Diagnosis and Management of Patients with SIHD. Fihn et al.**<br>**3.3.2 (pg e78)**<br>Coronary angiography is not recommended to further assess risk in patients with SIHD who have preserved LV function (EF [illegible]50%) and low-risk criteria on noninvasive testing.
	**IIb**	C	**2014 ACC/AHA Multi-Societal Focused Update of the Guideline for the Diagnosis and Management of Patients with SIHD. Fihn et al.**<br>**2.3 (pg e1753)**<br>Coronary angiography might be considered in patients with stress test results of acceptable quality that do not suggest the presence of CAD when clinical suspicion of CAD remains high and there is a high likelihood that the findings will result in important changes to therapy.
	**I**	C	**2014 ACC/AHA Multi-Societal Focused Update of the Guideline for the Diagnosis and Management of Patients with SIHD. Fihn et al.**<br>**2.3 (pg 1933)**<br>Coronary angiography is useful in patients with presumed SIHD who have unacceptable ischemic symptoms despite GDMT and who are amenable to, and candidates for, coronary revascularization.
14. CTCA with no CAD or up to 49% stenosis (CAD-RADS 0-2)	**IIa**	**C**	**2014 ACC/AHA Multi-Societal Focused Update of the Guideline for the Diagnosis and Management of Patients with SIHD. Fihn et al.**<br>**2.3 (pg 1933)**<br>Coronary angiography is reasonable in patients with suspected symptomatic SIHD who cannot undergo diagnostic stress testing, or have indeterminate or nondiagnostic stress tests, when there is a high likelihood that the findings will result in important changes to therapy.

15. CCTA with moderate stenosis 50-69% (CAD-RADS 3)	**I**<br><br>**I**<br><br>**IIa**	**B**<br><br>**C**<br><br>**C**	**2014 ACC/AHA Multi-Societal Focused Update of the Guideline for the Diagnosis and Management of Patients with SIHD. Fihn et al.**<br>**3.2.2.3 (pg e74)**<br>Either exercise or pharmacological stress with imaging (nuclear MPI, echocardiography, or CMR) is recommended for risk assessment in patients with SIHD who are being considered for revascularization of known coronary stenosis of unclear physiological significance.<br><br>**2014 ACC/AHA Multi-Societal Focused Update of the Guideline for the Diagnosis and Management of Patients with SIHD. Fihn et al.**<br>**2.3 (pg 1933)**<br>Coronary angiography is useful in patients with presumed SIHD who have unacceptable ischemic symptoms despite GDMT and who are amenable to, and candidates for, coronary revascularization.<br><br>**2014 ACC/AHA Multi-Societal Focused Update of the Guideline for the Diagnosis and Management of Patients with SIHD. Fihn et al.**<br>**2.3 (pg 1933)**<br>Coronary angiography is reasonable in patients with suspected symptomatic SIHD who cannot undergo diagnostic stress testing, or have indeterminate or nondiagnostic stress tests, when there is a high likelihood that the findings will result in important changes to therapy.
16. CTCA with severe stenosis >=70% (CAD-RADS 4-5)	**I**<br><br>**I**	**B**<br><br>**C**	**2014 ACC/AHA Multi-Societal Focused Update of the Guideline for the Diagnosis and Management of Patients with SIHD. Fihn et al.**<br>**3.2.2.3 (pg e74)**<br>Either exercise or pharmacological stress with imaging (nuclear MPI, echocardiography, or CMR) is recommended for risk assessment in patients with SIHD who are being considered for revascularization of known coronary stenosis of unclear physiological significance.

	IIa	C	**2014 ACC/AHA Multi-Societal Focused Update of the Guideline for the Diagnosis and Management of Patients with SIHD. Fihn et al.**<br>**3.3.2 (pg e78)**<br>Coronary arteriography is recommended for patients with SIHD whose clinical characteristics and results of noninvasive testing indicate a high likelihood of severe IHD and when the benefits are deemed to exceed risk.
	I	C	**2014 ACC/AHA Multi-Societal Focused Update of the Guideline for the Diagnosis and Management of Patients with SIHD. Fihn et al.**<br>**2.3 (pg e1753)**<br>Coronary angiography is reasonable to define the extent and severity of coronary artery disease (CAD) in patients with suspected SIHD whose clinical characteristics and results of noninvasive testing (exclusive of stress testing) indicate a high likelihood of severe IHD and who are amenable to, and candidates for, coronary revascularization.
	IIa	C	**2014 ACC/AHA Multi-Societal Focused Update of the Guideline for the Diagnosis and Management of Patients with SIHD. Fihn et al.**<br>**2.3 (pg 1933)**<br>Coronary angiography is useful in patients with presumed SIHD who have unacceptable ischemic symptoms despite GDMT and who are amenable to, and candidates for, coronary revascularization.
			**2014 ACC/AHA Multi-Societal Focused Update of the Guideline for the Diagnosis and Management of Patients with SIHD. Fihn et al.**<br>**2.3 (pg 1933)**<br>Coronary angiography is reasonable in patients with suspected symptomatic SIHD who cannot undergo diagnostic stress testing, or have indeterminate or nondiagnostic stress tests, when there is a high likelihood that the findings will result in important changes to therapy.

	**IIa**	**C**	**2014 ACC/AHA Multi-Societal Focused Update of the Guideline for the Diagnosis and Management of Patients with SIHD. Fihn et al.**<br>**3.3.2 (pg e78)**<br>Coronary angiography is reasonable to further assess risk in pa- tients with SIHD and inconclusive prognostic information after noninvasive testing or in patients for whom noninvasive testing is contraindicated or inadequate.
	**IIa**	**C**	**2014 ACC/AHA Multi-Societal Focused Update of the Guideline for the Diagnosis and Management of Patients with SIHD. Fihn et al.**<br>**2.3 (pg 1933)**<br>Coronary angiography is useful in patients with presumed SIHD who have unacceptable ischemic symptoms despite GDMT and who are amenable to, and candidates for, coronary revascularization.
17. CTCA inconclusive (CAD RADS N)	**IIa**	**C**	**2014 ACC/AHA Multi-Societal Focused Update of the Guideline for the Diagnosis and Management of Patients with SIHD. Fihn et al.**<br>**2.3 (pg 1933)**<br>Coronary angiography is reasonable in patients with suspected symptomatic SIHD who cannot undergo diagnostic stress testing, or have indeterminate or nondiagnostic stress tests, when there is a high likelihood that the findings will result in important changes to therapy.
	**III**	**B**	**2014 ACC/AHA Multi-Societal Focused Update of the Guideline for the Diagnosis and Management of Patients with SIHD. Fihn et al.**<br>**3.3.2 (pg e78)**<br>Coronary angiography is not recommended to further assess risk in patients with SIHD who have preserved LV function (EF 50%) and low-risk criteria on noninvasive testing.
18. CAC score = 0 (CAC-DRS 0)	**I**	**C**	**2014 ACC/AHA Multi-Societal Focused Update of the Guideline for the Diagnosis and Management of Patients with SIHD. Fihn et al.**

	IIa	C	**2.3 (pg 1933)**<br>Coronary angiography is useful in patients with presumed SIHD who have unacceptable ischemic symptoms despite GDMT and who are amenable to, and candidates for, coronary revascularization.<br><br>**2014 ACC/AHA Multi-Societal Focused Update of the Guideline for the Diagnosis and Management of Patients with SIHD. Fihn et al.**<br>**2.3 (pg 1933)**<br>Coronary angiography is reasonable in patients with suspected symptomatic SIHD who cannot undergo diagnostic stress testing, or have indeterminate or nondiagnostic stress tests, when there is a high likelihood that the findings will result in important changes to therapy.
19. CAC score 1-99 (CAC-DRS 1)			
20. CAC score 100-299 (CAC-DRS 2)			
21. CAC score ≥300 (CAC-DRS 3)			
22. Invasive coronary angiography with mild or no CAD and/or negative invasive physiological testing			
23. Invasive coronary angiography with intermediate severity and/or invasive physiological testing not done	I<br><br>IIa<br><br>IIa	B<br><br>A<br><br>B	**2014 ACC/AHA Multi-Societal Focused Update of the Guideline for the Diagnosis and Management of Patients with SIHD. Fihn et al.**<br>**3.2.2.3 (pg e74)**<br>Either exercise or pharmacological stress with imaging (nuclear MPI, echocardiography, or CMR) is recommended for risk assessment in patients with SIHD who are being considered for revascularization of known coronary stenosis of unclear physiological significance.<br><br>**2011 ACCF/AHA/SCAI PCI Guideline**<br>FFR is reasonable to assess angiographic intermediate coronary lesions (50% to 70% diameter stenosis) and can be useful for guiding revascularization decisions in patients with SIHD.<br><br>IVUS is reasonable for the assessment of angiographically indeterminant left main CAD.

	**IIb**	**C**	IVUS may be reasonable for the assessment of non–left main coronary arteries with angiographically intermediate coronary stenoses (50% to 70% diameter stenosis).
24. Invasive coronary angiography with obstructive CAD and/or abnormal invasive physiological testing			

References:

The following recommendations have been reviewed and could be applied to the clinical scenarios listed in Tables 1.2, 1.3 and 2.2 depending on the patient's history leading to the established diagnosis of stable ischemic heart disease.

2014 ACC/AHA Multi-Societal Focused Update of the Guideline for the Diagnosis and Management of Patients with SIHD. Fihn et al.
2.2.2.3 (pg e67), class IIa (1):
CCTA is reasonable for patients with an intermediate pretest probability of IHD who a) have continued symptoms with prior normal test findings, or b) have inconclusive results from prior exercise or pharmacological stress testing, or c) are unable to undergo stress with nuclear MPI or echocardiography. (Level of Evidence: C)

2014 ACC/AHA Multi-Societal Focused Update of the Guideline for the Diagnosis and Management of Patients with SIHD. Fihn et al.
3.2.2.1 (pg e74), class I (1):
Standard exercise ECG testing is recommended for risk assessment in patients with SIHD who are able to exercise to an adequate workload and have an interpretable ECG. (Level of Evidence: B)

2014 ACC/AHA Multi-Societal Focused Update of the Guideline for the Diagnosis and Management of Patients with SIHD. Fihn et al.
3.2.2.1 (pg e74), class I (2):
The addition of either nuclear MPI or echocardiography to standard exercise ECG testing is recommended for risk assessment in patients with SIHD who are able to exercise to an adequate workload but have an uninterpretable ECG not due to LBBB or ventricular pacing. (Level of Evidence: B)

2014 ACC/AHA Multi-Societal Focused Update of the Guideline for the Diagnosis and Management of Patients with SIHD. Fihn et al.
3.2.2.1 (pg e74), class IIa (1):
The addition of either nuclear MPI or echocardiography to standard exercise ECG testing is reasonable for risk assessment in patients with SIHD who are able to exercise to an adequate workload and have an interpretable ECG. (Level of Evidence: B)

2014 ACC/AHA Multi-Societal Focused Update of the Guideline for the Diagnosis and Management of Patients with SIHD. Fihn et al.
3.2.2.1 (pg e74), class IIa (2):
CMR with pharmacological stress is reasonable for risk assessment in patients with SIHD who are able to exercise to an adequate workload but have an uninterpretable ECG. (Level of Evidence: B)

2014 ACC/AHA Multi-Societal Focused Update of the Guideline for the Diagnosis and Management of Patients with SIHD. Fihn et al.
3.2.2.1 (pg e74), class IIb (1):
CCTA may be reasonable for risk assessment in patients with SIHD who are able to exercise to an adequate workload but have an uninterpretable ECG. (Level of Evidence: B)

2014 ACC/AHA Multi-Societal Focused Update of the Guideline for the Diagnosis and Management of Patients with SIHD. Fihn et al.
3.2.2.1 (pg e74), class III (1):
Pharmacological stress imaging (nuclear MPI, echocardiography, or CMR) or CCTA is not recommended for risk assessment in patients with SIHD who are able to exercise to an adequate workload and have an interpretable ECG. (Level of Evidence: C)

2014 ACC/AHA Multi-Societal Focused Update of the Guideline for the Diagnosis and Management of Patients with SIHD. Fihn et al.
3.2.2.2 (pg e74), class I (1):
Pharmacological stress with either nuclear MPI or echocardiography is recommended for risk assessment in patients with SIHD who are unable to exercise to an adequate workload regardless of interpretability of ECG. (Level of Evidence: B)

2014 ACC/AHA Multi-Societal Focused Update of the Guideline for the Diagnosis and Management of Patients with SIHD. Fihn et al.
3.2.2.2 (pg e74), class IIa (1):
Pharmacological stress CMR is reasonable for risk assessment in patients with SIHD who are unable to exercise to an adequate workload regardless of interpretability of ECG. (Level of Evidence: B)

2014 ACC/AHA Multi-Societal Focused Update of the Guideline for the Diagnosis and Management of Patients with SIHD. Fihn et al.
3.2.2.2 (pg e74), class IIa (2):
CCTA can be useful as a first-line test for risk assessment in patients with SIHD who are unable to exercise to an adequate workload regardless of interpretability of ECG (286). (Level of Evidence: C)

2014 ACC/AHA Multi-Societal Focused Update of the Guideline for the Diagnosis and Management of Patients with SIHD. Fihn et al.
3.2.2.3 (pg e74), class I (1):
Pharmacological stress with either nuclear MPI or echocardiography is recommended for risk assessment in patients with SIHD who have LBBB on ECG, regardless of ability to exercise to an adequate workload. (Level of Evidence: B)

2014 ACC/AHA Multi-Societal Focused Update of the Guideline for the Diagnosis and Management of Patients with SIHD. Fihn et al.
3.2.2.3 (pg e74), class I (2):
Either exercise or pharmacological stress with imaging (nuclear MPI, echocardiography, or CMR) is recommended for risk assessment in patients with SIHD who are being considered for revascularization of known coronary stenosis of unclear physiological significance. (Level of Evidence: B)

2014 ACC/AHA Multi-Societal Focused Update of the Guideline for the Diagnosis and Management of Patients with SIHD. Fihn et al.
3.2.2.3 (pg e75), class IIa (1):
CCTA can be useful for risk assessment in patients with SIHD who have an indeterminate result from functional testing. (Level of Evidence: C)

ACC/AHA/ASE/ASNC/ASPC/HFSA/HRS/SCAI/SCCT/SCMR/STS 2023 Multimodality Appropriate Use Criteria for the Detection and Risk Assessment of Chronic Coronary Disease: Guideline Mapping File

2014 ACC/AHA Multi-Societal Focused Update of the Guideline for the Diagnosis and Management of Patients with SIHD. Fihn et al.
3.2.2.3 (pg e75), class IIb (1):
CCTA might be considered for risk assessment in patients with SIHD unable to undergo stress imaging or as an alternative to invasive coronary angiography when functional testing indicates a moderate- to high-risk result and knowledge of angiographic coronary anatomy is unknown. (Level of Evidence: C)

2014 ACC/AHA Multi-Societal Focused Update of the Guideline for the Diagnosis and Management of Patients with SIHD. Fihn et al.
3.2.2.3 (pg e75), class III (1):
A request to perform either a) more than 1 stress imaging study or b) a stress imaging study and a CCTA at the same time is not recommended for risk assessment in patients with SIHD. (Level of Evidence: C)

2014 ACC/AHA Multi-Societal Focused Update of the Guideline for the Diagnosis and Management of Patients with SIHD. Fihn et al.
3.3.1 (pg e78), class I (1):
Patients **with SIHD** who have survived sudden cardiac death or potentially life-threatening ventricular arrhythmia should undergo coronary angiography to assess cardiac risk. (Level of Evidence: B)

2014 ACC/AHA Multi-Societal Focused Update of the Guideline for the Diagnosis and Management of Patients with SIHD. Fihn et al.
3.3.1 (pg e78), class I (2):
Patients with SIHD who develop symptoms and signs of heart failure should be evaluated to determine whether coronary angiography should be performed for risk assessment. (Level of Evidence: B)

2014 ACC/AHA Multi-Societal Focused Update of the Guideline for the Diagnosis and Management of Patients with SIHD. Fihn et al.
3.3.2 (pg e78), class I (1):
Coronary arteriography is recommended for patients with SIHD whose clinical characteristics and results of noninvasive testing indicate a high likelihood of severe IHD and when the benefits are deemed to exceed risk. (Level of Evidence: C)

2014 ACC/AHA Multi-Societal Focused Update of the Guideline for the Diagnosis and Management of Patients with SIHD. Fihn et al.
3.3.2 (pg e78), class IIa (1):
Coronary angiography is reasonable to further assess risk in patients with SIHD who have depressed LV function (EF [illegible]50%) and moderate risk criteria on noninvasive testing with demonstrable ischemia. (Level of Evidence: C)

2014 ACC/AHA Multi-Societal Focused Update of the Guideline for the Diagnosis and Management of Patients with SIHD. Fihn et al.
3.3.2 (pg e78), class IIa (2):
Coronary angiography is reasonable to further assess risk in patients with SIHD and inconclusive prognostic information after noninvasive testing or in patients for whom noninvasive testing is contraindicated or inadequate. (Level of Evidence: C)

2014 ACC/AHA Multi-Societal Focused Update of the Guideline for the Diagnosis and Management of Patients with SIHD. Fihn et al.
3.3.2 (pg e78), class IIa (3):
Coronary angiography for risk assessment is reasonable for patients with SIHD who have unsatisfactory quality of life due to angina, have preserved LV function (EF [illegible]50%), and have intermediate risk criteria on noninvasive testing. (Level of Evidence: C)

2014 ACC/AHA Multi-Societal Focused Update of the Guideline for the Diagnosis and Management of Patients with SIHD. Fihn et al.
3.3.2 (pg e78), class III (1):
Coronary angiography for risk assessment is not recommended in patients with SIHD who elect not to undergo revascularization or who are not candidates for revascularization because of comorbidities or individual preferences. (Level of Evidence: B)

2014 ACC/AHA Multi-Societal Focused Update of the Guideline for the Diagnosis and Management of Patients with SIHD. Fihn et al.
3.3.2 (pg e78), class III (2):
Coronary angiography is not recommended to further assess risk in patients with SIHD who have preserved LV function (EF 50%) and low-risk criteria on noninvasive testing. (Level of Evidence: B)

2014 ACC/AHA Multi-Societal Focused Update of the Guideline for the Diagnosis and Management of Patients with SIHD. Fihn et al.
6.3.2 (pg e124), class IIa (1):
Nuclear MPI, echocardiography, or CMR with either exercise or pharmacological stress can be useful for follow-up assessment at 2-year or longer intervals in patients with SIHD with prior evidence of silent ischemia or who are at high risk for a recurrent cardiac event and a) are unable to exercise to an adequate workload, b) have an uninterpretable ECG, or c) have a history of incomplete coronary revascularization. (Level of Evidence: C)

2014 ACC/AHA Multi-Societal Focused Update of the Guideline for the Diagnosis and Management of Patients with SIHD. Fihn et al.
6.3.2 (pg e124), class IIb (1):
Standard exercise ECG testing performed at 1-year or longer inter- vals might be considered for follow-up assessment in patients with SIHD who have had prior evidence of silent ischemia or are at high risk for a recurrent cardiac event and are able to exercise to an adequate workload and have an interpretable ECG. (Level of Evidence: C)

2014 ACC/AHA Multi-Societal Focused Update of the Guideline for the Diagnosis and Management of Patients with SIHD. Fihn et al.
6.3.2 (pg e124), class IIb (2):
In patients who have no new or worsening symptoms or no prior evidence of silent ischemia and are not at high risk for a recurrent cardiac event, the usefulness of annual surveillance exercise ECG testing is not well established. (Level of Evidence: C)

2014 ACC/AHA Multi-Societal Focused Update of the Guideline for the Diagnosis and Management of Patients with SIHD. Fihn et al.
6.3.2 (pg e124), class III (1):
Nuclear MPI, echocardiography, or CMR, with either exercise or pharmacological stress or CCTA, is not recommended for follow-up assessment in patients with SIHD, if performed more frequently than at a) 5-year intervals after CABG or b) 2-year intervals after PCI (10,12,15). (Level of Evidence: C)

Role of noninvasive testing in the clinical evaluation of women with suspected ischemic heart disease: a consensus statement from the American Heart Association. Mieres JH, et al. Circulation. 2014 Jul 22;130(4):350-79. doi: 10.1161/CIR.0000000000000061. Epub 2014 Jun 16

Stress echocardiography is recommended for identification of obstructive CAD and estimation of prognosis for symptomatic women at intermediate-high IHD risk and with any of the following: (a) resting ST-segment abnormalities, (b) functional disability, or (c) indeterminate or intermediate-risk stress ECG *(Class I; Level of Evidence B)*.

For the premenopausal woman with functional disability, pharmacological stress echocardiography is recommended for identification of obstructive CAD and estimation of prognosis *(Class I; Level of Evidence C*).

The following recommendations have been reviewed and could be applied to the clinical scenarios listed in Tables 1.2 and 1.3 depending on the patient's history leading to the established diagnosis of stable ischemic heart disease.
2014 ACC/AHA Multi-Societal Focused Update of the Guideline for the Diagnosis and Management of Patients with SIHD. Fihn et al.
6.3.1.1 (pg e122), class I (1):
Standard exercise ECG testing is recommended in patients with known SIHD who have new or worsening symptoms not consistent with UA and who have a) at least moderate physical functioning and no disabling comorbidity and b) an interpretable ECG. (Level of Evidence: B)

2014 ACC/AHA Multi-Societal Focused Update of the Guideline for the Diagnosis and Management of Patients with SIHD. Fihn et al.
6.3.1.1 (pg e123), class I (2):
Exercise with nuclear MPI or echocardiography is recommended in patients with known SIHD who have new or worsening symptoms not consistent with UA and who have a) at least moderate physical functioning or no disabling comorbidity but b) an uninterpretable ECG. (Level of Evidence: B)

2014 ACC/AHA Multi-Societal Focused Update of the Guideline for the Diagnosis and Management of Patients with SIHD. Fihn et al.
6.3.1.1 (pg e123), class II (1):
Exercise with nuclear MPI or echocardiography is reasonable in patients with known SIHD who have new or worsening symptoms not consistent with UA and who have a) at least moderate physical functioning and no disabling comorbidity, b) previously required imaging with exercise stress, or c) known multivessel disease or high risk for multivessel disease. (Level of Evidence: B)

2014 ACC/AHA Multi-Societal Focused Update of the Guideline for the Diagnosis and Management of Patients with SIHD. Fihn et al.
6.3.1.1 (pg e123), class III (1):
Pharmacological stress imaging with nuclear MPI, echocardiography, or CMR is not recommended in patients with known SIHD who have new or worsening symptoms not consistent with UA and who are capable of at least moderate physical functioning or have no disabling comorbidity (333). (Level of Evidence: C)

2014 ACC/AHA Multi-Societal Focused Update of the Guideline for the Diagnosis and Management of Patients with SIHD. Fihn et al.
6.3.1.2 (pg e123), class I (1):
Pharmacological stress imaging with nuclear MPI or echocardiography is recommended in patients with known SIHD who have new or worsening symptoms not consistent with UA and who are incapable of at least moderate physical functioning or have disabling comorbidity. (Level of Evidence: B)

2014 ACC/AHA Multi-Societal Focused Update of the Guideline for the Diagnosis and Management of Patients with SIHD. Fihn et al.
6.3.1.2 (pg e123), class IIa (1):
Pharmacological stress imaging with CMR is reasonable in patients with known SIHD who have new or worsening symptoms not consistent with UA and who are incapable of at least moderate physical functioning or have disabling comorbidity. (Level of Evidence: B)

2014 ACC/AHA Multi-Societal Focused Update of the Guideline for the Diagnosis and Management of Patients with SIHD. Fihn et al.
6.3.1.2 (pg e123-124), class III (1):

Standard exercise ECG testing should not be performed in patients with known SIHD who have new or worsening symptoms not consistent with UA and who a) are incapable of at least moderate physical functioning or have disabling comorbidity or b) have an uninterpretable ECG. (Level of Evidence: C)

2014 ACC/AHA Multi-Societal Focused Update of the Guideline for the Diagnosis and Management of Patients with SIHD. Fihn et al.
6.3.1.3 (pg e124), class IIb (2):
CCTA might be reasonable in patients with known SIHD who have new or worsening symptoms not consistent with UA, irrespective of ability to exercise, in the absence of known moderate or severe calcification or if the CCTA is intended to assess coronary stents less than 3 mm in diameter. (Level of Evidence: B)

2014 ACC/AHA Multi-Societal Focused Update of the Guideline for the Diagnosis and Management of Patients with SIHD. Fihn et al.
6.3.1.3 (pg e124), class III (1):
CCTA should not be performed for assessment of native coronary arteries with known moderate or severe calcification or with coronary stents less than 3 mm in diameter in patients with known SIHD who have new or worsening symptoms not consistent with UA, irrespective of ability to exercise. (Level of Evidence: B)

2014 ACC/AHA Multi-Societal Focused Update of the Guideline for the Diagnosis and Management of Patients with SIHD. Fihn et al.
2.3 (pg e1753), class IIa (1)
Coronary angiography is reasonable to define the extent and severity of coronary artery disease (CAD) in patients with suspected SIHD whose clinical characteristics and results of noninvasive testing (exclusive of stress testing) indicate a high likelihood of severe IHD and who are amenable to, and candidates for, coronary revascularization. (Level of Evidence: C)

2014 ACC/AHA Multi-Societal Focused Update of the Guideline for the Diagnosis and Management of Patients with SIHD. Fihn et al.
2.3 (pg e1753), class IIa (2)
Coronary angiography is reasonable in patients with suspected symptomatic SIHD who cannot undergo diagnostic stress testing, or have indeterminate or nondiagnostic stress tests, when there is a high likelihood that the findings will result in important changes to therapy. (Level of Evidence: C)

2014 ACC/AHA Multi-Societal Focused Update of the Guideline for the Diagnosis and Management of Patients with SIHD. Fihn et al.
2.3 (pg e1753), class IIb (1)
Coronary angiography might be considered in patients with stress test results of acceptable quality that do not suggest the presence of CAD when clinical suspicion of CAD remains high and there is a high likelihood that the findings will result in important changes to therapy (Level of Evidence: C).

2014 ACC/AHA Multi-Societal Focused Update of the Guideline for the Diagnosis and Management of Patients with SIHD. Fihn et al.
2.3 (pg 1933), Class I
Coronary angiography is useful in patients with presumed SIHD who have unacceptable ischemic symptoms despite GDMT and who are amenable to, and candidates for, coronary revascularization. (Level of Evidence: C)

2014 ACC/AHA Multi-Societal Focused Update of the Guideline for the Diagnosis and Management of Patients with SIHD. Fihn et al.
2.3 (pg 1933), Class IIa
Coronary angiography is reasonable to define the extent and severity of coronary artery disease (CAD) in patients with suspected SIHD whose clinical characteristics and results of noninvasive testing (exclusive of stress testing) indicate a high likelihood of severe IHD and who are amenable to, and candidates for, coronary revascularization (7–12). (Level of Evidence: C)

2014 ACC/AHA Multi-Societal Focused Update of the Guideline for the Diagnosis and Management of Patients with SIHD. Fihn et al.
2.3 (pg 1933), Class IIa
Coronary angiography is reasonable in patients with suspected symptomatic SIHD who cannot undergo diagnostic stress testing, or have indeterminate or nondiagnostic stress tests, when there is a high likelihood that the findings will result in important changes to therapy. (Level of Evidence: C)

2014 ACC/AHA Multi-Societal Focused Update of the Guideline for the Diagnosis and Management of Patients with SIHD. Fihn et al.
2.3 (pg 1933), Class IIb
Coronary angiography might be considered in patients with stress test results of acceptable quality that do not suggest the presence of CAD when clinical suspicion of CAD remains high and there is a high likelihood that the findings will result in important changes to therapy. (Level of Evidence: C)

Table 1.3 Symptomatic Patients With Prior MI or Revascularization

Indication	Class of Rec. (COR)	Level of Evidence (LEVEL) Review	Reference
25. Incomplete revascularization	IIa	C	**2014 ACC/AHA Multi-Societal Focused Update of the Guideline for the Diagnosis and Management of Patients with SIHD. Fihn et al. 6.3.2 (pg e124)**

			Nuclear MPI, echocardiography, or CMR with either exercise or pharmacological stress can be useful for follow-up assessment at 2-year or longer intervals in patients with SIHD with prior evidence of silent ischemia or who are at high risk for a recurrent cardiac event and a) are unable to exercise to an adequate workload, b) have an uninterpretable ECG, or c) have a history of incomplete coronary revascularization.<br><br>**2014 ACC/AHA Multi-Societal Focused Update of the Guideline for the Diagnosis and Management of Patients with SIHD. Fihn et al.**<br>**2.3 (pg 1933)**<br>Coronary angiography is useful in patients with presumed SIHD who have unacceptable ischemic symptoms despite GDMT and who are amenable to, and candidates for, coronary revascularization.<br><br>**2014 ACC/AHA Multi-Societal Focused Update of the Guideline for the Diagnosis and Management of Patients with SIHD. Fihn et al.**<br>**2.3 (pg 1933)**<br>Coronary angiography is reasonable to define the extent and severity of coronary artery disease (CAD) in patients with suspected SIHD whose clinical characteristics and results of noninvasive testing (exclusive of stress testing) indicate a high likelihood of severe IHD and who are amenable to, and candidates for, coronary revascularization (7–12).<br><br>**2014 ACC/AHA Multi-Societal Focused Update of the Guideline for the Diagnosis and Management of Patients with SIHD. Fihn et al.**<br>**2.3 (pg 1933)**<br>Coronary angiography is reasonable in patients with suspected symptomatic SIHD who cannot undergo diagnostic stress testing, or have indeterminate or nondiagnostic stress tests, when there is a high likelihood that the findings will result in important changes to therapy.<br><br>**2014 ACC/AHA Multi-Societal Focused Update of the Guideline for the Diagnosis and Management of Patients with SIHD. Fihn et al.**<br>**2.3 (pg 1933)**<br>Coronary angiography might be considered in patients with stress test results of acceptable quality that do not suggest the presence of CAD when clinical suspicion of CAD remains high and there is a high likelihood that the findings will result in important changes to therapy.
	**I**<br><br>**IIa**<br><br>**IIa**<br><br>**IIb**	**C**<br><br>**C**<br><br>**C**<br><br>**C**	

	I	C	**2014 ACC/AHA Multi-Societal Focused Update of the Guideline for the Diagnosis and Management of Patients with SIHD. Fihn et al.**<br>**2.3 (pg 1933)**<br>Coronary angiography is useful in patients with presumed SIHD who have unacceptable ischemic symptoms despite GDMT and who are amenable to, and candidates for, coronary revascularization.
	IIa	C	**2014 ACC/AHA Multi-Societal Focused Update of the Guideline for the Diagnosis and Management of Patients with SIHD. Fihn et al.**<br>**2.3 (pg 1933)**<br>Coronary angiography is reasonable to define the extent and severity of coronary artery disease (CAD) in patients with suspected SIHD whose clinical characteristics and results of noninvasive testing (exclusive of stress testing) indicate a high likelihood of severe IHD and who are amenable to, and candidates for, coronary revascularization (7–12).
	IIa	C	**2014 ACC/AHA Multi-Societal Focused Update of the Guideline for the Diagnosis and Management of Patients with SIHD. Fihn et al.**<br>**2.3 (pg 1933)**<br>Coronary angiography is reasonable in patients with suspected symptomatic SIHD who cannot undergo diagnostic stress testing, or have indeterminate or nondiagnostic stress tests, when there is a high likelihood that the findings will result in important changes to therapy
26. Prior PCI, symptoms similar to prior ischemic episode and/or anginal symptoms	IIb	C	**2014 ACC/AHA Multi-Societal Focused Update of the Guideline for the Diagnosis and Management of Patients with SIHD. Fihn et al.**<br>**2.3 (pg 1933)**<br>Coronary angiography might be considered in patients with stress test results of acceptable quality that do not suggest the presence of CAD when clinical suspicion of CAD remains high and there is a high likelihood that the findings will result in important changes to therapy.
27. Prior PCI, nonanginal symptoms			
28. Prior CABG, symptoms similar to prior ischemic episode and/or anginal symptoms	I	C	**2014 ACC/AHA Multi-Societal Focused Update of the Guideline for the Diagnosis and Management of Patients with SIHD. Fihn et al.**<br>**2.3 (pg 1933)**

	**IIa**	**C**	Coronary angiography is useful in patients with presumed SIHD who have unacceptable ischemic symptoms despite GDMT and who are amenable to, and candidates for, coronary revascularization.
	**IIa**	**C**	**2014 ACC/AHA Multi-Societal Focused Update of the Guideline for the Diagnosis and Management of Patients with SIHD. Fihn et al. 2.3 (pg 1933)** Coronary angiography is reasonable to define the extent and severity of coronary artery disease (CAD) in patients with suspected SIHD whose clinical characteristics and results of noninvasive testing (exclusive of stress testing) indicate a high likelihood of severe IHD and who are amenable to, and candidates for, coronary revascularization (7–12).
	**IIb**	**C**	**2014 ACC/AHA Multi-Societal Focused Update of the Guideline for the Diagnosis and Management of Patients with SIHD. Fihn et al. 2.3 (pg 1933)** Coronary angiography is reasonable in patients with suspected symptomatic SIHD who cannot undergo diagnostic stress testing, or have indeterminate or nondiagnostic stress tests, when there is a high likelihood that the findings will result in important changes to therapy. <br>**2014 ACC/AHA Multi-Societal Focused Update of the Guideline for the Diagnosis and Management of Patients with SIHD. Fihn et al. 2.3 (pg 1933)** Coronary angiography might be considered in patients with stress test results of acceptable quality that do not suggest the presence of CAD when clinical suspicion of CAD remains high and there is a high likelihood that the findings will result in important changes to therapy.
29. Prior CABG, nonanginal symptoms			
30. Prior MI, no revascularization, symptoms similar to prior ischemic episode and/or anginal	**I** <br>IIa	**C** <br>**C**	**2014 ACC/AHA Multi-Societal Focused Update of the Guideline for the Diagnosis and Management of Patients with SIHD. Fihn et al. 2.3 (pg 1933)** Coronary angiography is useful in patients with presumed SIHD who have unacceptable ischemic symptoms despite GDMT and who are amenable to, and candidates for, coronary revascularization.

			**2014 ACC/AHA Multi-Societal Focused Update of the Guideline for the Diagnosis and Management of Patients with SIHD. Fihn et al.**<br>**2.3 (pg 1933)**<br>Coronary angiography is reasonable to define the extent and severity of coronary artery disease (CAD) in patients with suspected SIHD whose clinical characteristics and results of noninvasive testing (exclusive of stress testing) indicate a high likelihood of severe IHD and who are amenable to, and candidates for, coronary revascularization (7–12).
	**IIa**	**C**	**2014 ACC/AHA Multi-Societal Focused Update of the Guideline for the Diagnosis and Management of Patients with SIHD. Fihn et al.**<br>**2.3 (pg 1933)**<br>Coronary angiography is reasonable in patients with suspected symptomatic SIHD who cannot undergo diagnostic stress testing, or have indeterminate or nondiagnostic stress tests, when there is a high likelihood that the findings will result in important changes to therapy.
	**IIb**	**C**	**2014 ACC/AHA Multi-Societal Focused Update of the Guideline for the Diagnosis and Management of Patients with SIHD. Fihn et al.**<br>**2.3 (pg 1933)**<br>Coronary angiography might be considered in patients with stress test results of acceptable quality that do not suggest the presence of CAD when clinical suspicion of CAD remains high and there is a high likelihood that the findings will result in important changes to therapy.
31. Prior MI, no revascularization, nonanginal symptoms	**I**	**C**	**2014 ACC/AHA Multi-Societal Focused Update of the Guideline for the Diagnosis and Management of Patients with SIHD. Fihn et al.**<br>**2.3 (pg 1933)**<br>Coronary angiography is useful in patients with presumed SIHD who have unacceptable ischemic symptoms despite GDMT and who are amenable to, and candidates for, coronary revascularization.
	**IIa**	**C**	**2014 ACC/AHA Multi-Societal Focused Update of the Guideline for the Diagnosis and Management of Patients with SIHD. Fihn et al.**<br>**2.3 (pg 1933)**<br>Coronary angiography is reasonable to define the extent and severity of coronary artery disease (CAD) in patients with suspected SIHD whose clinical characteristics and results of noninvasive testing (exclusive of stress testing) indicate a high likelihood of severe IHD and who are amenable to, and candidates for, coronary revascularization (7–12).

	**IIa**	**C**	**2014 ACC/AHA Multi-Societal Focused Update of the Guideline for the Diagnosis and Management of Patients with SIHD. Fihn et al.**<br>**2.3 (pg 1933)**<br>Coronary angiography is reasonable in patients with suspected symptomatic SIHD who cannot undergo diagnostic stress testing, or have indeterminate or nondiagnostic stress tests, when there is a high likelihood that the findings will result in important changes to therapy.
	**IIb**	**C**	**2014 ACC/AHA Multi-Societal Focused Update of the Guideline for the Diagnosis and Management of Patients with SIHD. Fihn et al.**<br>**2.3 (pg 1933)**<br>Coronary angiography might be considered in patients with stress test results of acceptable quality that do not suggest the presence of CAD when clinical suspicion of CAD remains high and there is a high likelihood that the findings will result in important changes to therapy.
32. Assessment of myocardial viability	**IIa**	**B**	**2013 Yancy et al. ACCF/AHA Heart Failure Guideline:**<br>**6.4 (pg e166)**<br>Viability assessment is reasonable in select situations when planning revascularization in HF patients with CAD (281–285).
33. Prior to cardiac rehabilitation, coronary disease (no new or worsening symptoms)			

References:
The following recommendations have been reviewed and could be applied to the clinical scenarios listed in Tables 1.3 depending on the patient’s symptoms and specific history of revascularization.

2014 ACC/AHA Multi-Societal Focused Update of the Guideline for the Diagnosis and Management of Patients with SIHD. Fihn et al.
6.3.1.3 (pg e124), class IIb (1):
CCTA for assessment of patency of CABG or of coronary stents 3 mm or larger in diameter might be reasonable in patients with known SIHD who have new or worsening symptoms not consistent with UA, irrespective of ability to exercise (1244–1248). (Level of Evidence: B)

2014 ACC/AHA Multi-Societal Focused Update of the Guideline for the Diagnosis and Management of Patients with SIHD. Fihn et al.
6.3.1.3 (pg e124), class IIb (2):
CCTA might be reasonable in patients with known SIHD who have new or worsening symptoms not consistent with UA, irrespective of ability to exercise, in the absence of known moderate or severe calcification or if the CCTA is intended to assess coronary stents less than 3 mm in diameter. (Level of Evidence: B)

2014 ACC/AHA Multi-Societal Focused Update of the Guideline for the Diagnosis and Management of Patients with SIHD. Fihn et al.
6.3.1.3 (pg e124), class III (1):
CCTA should not be performed for assessment of native coronary arteries with known moderate or severe calcification or with coronary stents less than 3 mm in diameter in patients with known SIHD who have new or worsening symptoms not consistent with UA, irrespective of ability to exercise. (Level of Evidence: B)

Table 2.1 Asymptomatic Patients Without Known ASCVD

Indication	Class of Rec. (COR)	Level of Evidence (LEVEL) Review	Reference
34. Low ASCVD risk < 5%*			
35. Borderline ASCVD risk 5% to 7.5%		N	**2013 ACC/AHA Guideline on the Assessment of Cardiovascular Risk. Goff et al. (pg 2945)**<br>Recommendation 3. The contribution of ApoB, chronic kidney disease, albuminuria, and cardiorespiratory fitness to risk assessment for a first ASCVD event is uncertain at present.<br>NHLBI Grade: N (No recommendation for or against)
36. Borderline ASCVD risk 5-7.5% with risk-enhancing factors			
37. Intermediate ASCVD risk 7.5-20% with or without risk-enhancing factors**			

38. High ASCVD risk >20%			

* Risk calculated using the ASCVD risk estimator
**Table C, Risk-Enhancing Factors

Role of noninvasive testing in the clinical evaluation of women with suspected ischemic heart disease: a consensus statement from the American Heart Association. Mieres JH, et al. Circulation. 2014 Jul 22;130(4):350-79. doi: 10.1161/CIR.0000000000000061. Epub 2014 Jun 16

Stress echocardiography is recommended for identification of obstructive CAD and estimation of prognosis for symptomatic women at intermediate-high IHD risk and with any of the following: (a) resting ST-segment abnormalities, (b) functional disability, or (c) indeterminate or intermediate-risk stress ECG *(Class I; Level of Evidence B)*.

For the premenopausal woman with functional disability, pharmacological stress echocardiography is recommended for identification of obstructive CAD and estimation of prognosis *(Class I; Level of Evidence C)*.

Table 2.2 Asymptomatic Patients With Prior Revascularization or MI

Indication	Class of Rec. (COR)	Level of Evidence (LEVEL) Review	Reference
39. Incomplete Revascularization	**IIa**	**C**	**2014 ACC/AHA Multi-Societal Focused Update of the Guideline for the Diagnosis and Management of Patients with SIHD. Fihn et al.<br>6.3.2 (pg e124)**

			Nuclear MPI, echocardiography, or CMR with either exercise or pharmacological stress can be useful for follow-up assessment at 2-year or longer intervals in patients with SIHD with prior evidence of silent ischemia or who are at high risk for a recurrent cardiac event and a) are unable to exercise to an adequate workload, b) have an uninterpretable ECG, or c) have a history of incomplete coronary revascularization.
40. Prior high-risk PCI			
41. < 5 years after CABG	**III**	**C**	**2014 ACC/AHA Multi-Societal Focused Update of the Guideline for the Diagnosis and Management of Patients with SIHD. Fihn et al.**<br>**6.3.2 (pg e124)**<br>Nuclear MPI, echocardiography, or CMR, with either exercise or pharmacological stress or CCTA, is not recommended for follow-up assessment in patients with SIHD, if performed more frequently than at a**)** 5-year intervals after CABG or b) 2-year intervals after PCI (10,12,15).
42. > 5 years after CABG	**I**<br>**I**	**C**<br>**C**	**2015 Eligibility and Disqualification Recommendations for Competitive Athletes With Cardiovascular Abnormalities**<br>Thompson PD, Myerburg RJ, Levine BD, et al. Eligibility and disqualification recommendations for competitive athletes with cardiovascular abnormalities.<br>Task Force 8: coronary artery disease: a scientific statement from the American Heart Association and American College of Cardiology. Circulation. 2015;132:e310–e314.<br>**Page e311**<br>Athletes with ASCAD should undergo maximal exercise testing to evaluate exercise tolerance, the presence of inducible ischemia, and the presence of exercise induced electrical instability. Testing should be performed on the subject's standard medical regimen, including b-adrenergic blocking medications
43. < 2 years after PCI	**III**<br>**III**	**C**<br>**C**	**2014 ACC/AHA Multi-Societal Focused Update of the Guideline for the Diagnosis and Management of Patients with SIHD. Fihn et al.**<br>**6.3.2 (pg e124)**<br>Nuclear MPI, echocardiography, or CMR, with either exercise or pharmacological stress or CCTA, is not recommended for follow-up assessment in patients with SIHD, if performed more frequently than at a**)** 5-year intervals after CABG or b) 2-year intervals after PCI (10,12,15).<br><br>**2011 ACCF/AHA/SCAI PCI Guideline. Levine et al.**<br>**6.4.1. Exercise Testing (pg e88)**

			Routine periodic stress testing of asymptomatic patients after PCI without specific clinical indications should not be performed.
44. > 2 years after PCI	III<br><br>I	C<br><br>C	**2011 ACCF/AHA/SCAI PCI Guideline. Levine et al.**<br>**6.4.1. Exercise Testing (pg e88)**<br>Routine periodic stress testing of asymptomatic patients after PCI without specific clinical indications should not be performed.<br><br>**2015 Eligibility and Disqualification Recommendations for Competitive Athletes With Cardiovascular Abnormalities**<br>Thompson PD, Myerburg RJ, Levine BD, et al. Eligibility and disqualification recommendations for competitive athletes with cardiovascular abnormalities.<br>Task Force 8: coronary artery disease: a scientific statement from the American Heart Association and American College of Cardiology. Circulation. 2015;;132:e310–e314.<br>**Page e311**<br>Athletes with ASCAD should undergo maximal exercise testing to evaluate exercise tolerance, the presence of inducible ischemia, and the presence of exercise induced electrical instability. Testing should be performed on the subject's standard medical regimen, including b-adrenergic blocking medications
45. Patients at high risk for or history of silent ischemia*	IIa<br><br>IIb	C<br><br>C	**2014 ACC/AHA Multi-Societal Focused Update of the Guideline for the Diagnosis and Management of Patients with SIHD. Fihn et al.**<br>**6.3.2 (pg e124)**<br>Nuclear MPI, echocardiography, or CMR with either exercise or pharmacological stress can be useful for follow-up assessment at 2-year or longer intervals in patients with SIHD with prior evidence of silent ischemia or who are at high risk for a recurrent cardiac event and a) are unable to exercise to an adequate workload, b) have an uninterpretable ECG, or c) have a history of incomplete coronary revascularization.<br><br>**2014 ACC/AHA Multi-Societal Focused Update of the Guideline for the Diagnosis and Management of Patients with SIHD. Fihn et al.**<br>**6.3.2 (pg e124)**<br>Standard exercise ECG testing performed at 1-year or longer inter- vals might be considered for follow-up assessment in patients with SIHD who have had prior evidence of silent ischemia or are at high risk for a recurrent cardiac event and are able to exercise to an adequate workload and have an interpretable ECG.

	IIa	C	**2014 ACC/AHA Multi-Societal Focused Update of the Guideline for the Diagnosis and Management of Patients with SIHD. Fihn et al.**<br>**2.3 (pg 1933)**<br>Coronary angiography is reasonable to define the extent and severity of coronary artery disease (CAD) in patients with suspected SIHD whose clinical characteristics and results of noninvasive testing (exclusive of stress testing) indicate a high likelihood of severe IHD and who are amenable to, and candidates for, coronary revascularization (7–12).
	IIa	C	**2014 ACC/AHA Multi-Societal Focused Update of the Guideline for the Diagnosis and Management of Patients with SIHD. Fihn et al.**<br>**2.3 (pg 1933)**<br>Coronary angiography is reasonable in patients with suspected symptomatic SIHD who cannot undergo diagnostic stress testing, or have indeterminate or nondiagnostic stress tests, when there is a high likelihood that the findings will result in important changes to therapy.
	IIb	C	**2014 ACC/AHA Multi-Societal Focused Update of the Guideline for the Diagnosis and Management of Patients with SIHD. Fihn et al.**<br>**2.3 (pg 1933)**<br>Coronary angiography might be considered in patients with stress test results of acceptable quality that do not suggest the presence of CAD when clinical suspicion of CAD remains high and there is a high likelihood that the findings will result in important changes to therapy.
46. Assessment of myocardial viability	IIa	C	**2013 ACCF/AHA Heart Failure Guideline. Yancy et al.**<br>**6.4 (pg e166)**<br>Noninvasive imaging to detect myocardial ischemia and viability is reasonable in patients presenting with de novo HF, who have known CAD and no angina, unless the patient is not eligible for revascularization of any kind.
	IIa	B	**2013 ACCF/AHA Heart Failure Guideline. Yancy et al.**<br>**6.4 (pg e166)**<br>Viability assessment is reasonable in select situations when planning revascularization in HF patients with CAD.
47. Isolated evaluation of bypass graft patency			

*Diabetes mellitus with accelerated progression of CAD, chronic kidney disease, peripheral artery disease, prior brachytherapy, in-stent restenosis, saphenous vein graft intervention

Table 2.3 Asymptomatic Patients Undergoing Assessment Prior to an Exercise Program or Cardiac Rehab

Indication		Level of Evidence (LEVEL) Review	Reference
48. Prior to initiation of an unsupervised exercise program, without known CCD	**I**	**C**	**2015 Eligibility and Disqualification Recommendations for Competitive Athletes With Cardiovascular Abnormalities**<br>Thompson PD, Myerburg RJ, Levine BD, et al. Eligibility and disqualification recommendations for competitive athletes with cardiovascular abnormalities. Task Force 8: coronary artery disease: a scientific statement from the American Heart Association and American College of Cardiology. Circulation. 2015;;132:e310–e314.<br>**Page e311**<br>Athletes with ASCAD should undergo maximal exercise testing to evaluate exercise tolerance, the presence of inducible ischemia, and the presence of exercise induced electrical instability. Testing should be performed on the subject's standard medical regimen, including b-adrenergic blocking medications
49. Prior to initiation of an unsupervised exercise program, with known CCD	**IIa**<br><br>**IIa**	**C**<br><br>**C**	**2011 ACCF/AHA/SCAI PCI Guideline. Levine et al.**<br>**6.4.1. Exercise Testing (pg e88)**<br>In patients entering a formal cardiac rehabilitation program after PCI, treadmill exercise testing is reasonable.<br><br>**2011 ACCF/AHA/SCAI PCI Guideline. Levine et al.**<br>**(e88)**<br>**6.4.1. Exercise Testing: Recommendations**<br>1. In patients entering a formal cardiac rehabilitation program after PCI, treadmill exercise testing is reasonable.

50. Prior to Cardiac Rehabilitation	**IIa**	**C**	**2011 ACCF/AHA/SCAI PCI Guideline. Levine et al.**<br>**6.4.1. Exercise Testing (pg e88)**<br>In patients entering a formal cardiac rehabilitation program after PCI, treadmill exercise testing is reasonable.

The following recommendation has been reviewed and could be applied to the clinical scenarios listed in Table 2.1 and 2.2.

2014 ACC/AHA Multi-Societal Focused Update of the Guideline for the Diagnosis and Management of Patients with SIHD. Fihn et al.
3.3.2 (pg e78), class III (4):
Coronary angiography is not recommended to assess risk in asymptomatic patients with no evidence of ischemia on noninvasive testing. (Level of Evidence: C)

2014 ACC/AHA Multi-Societal Focused Update of the Guideline for the Diagnosis and Management of Patients with SIHD. Fihn et al.
2.3 (pg 1933), Class IIa
Coronary angiography is reasonable to define the extent and severity of coronary artery disease (CAD) in patients with suspected SIHD whose clinical characteristics and results of noninvasive testing (exclusive of stress testing) indicate a high likelihood of severe IHD and who are amenable to, and candidates for, coronary revascularization (7–12). (Level of Evidence: C)

2014 ACC/AHA Multi-Societal Focused Update of the Guideline for the Diagnosis and Management of Patients with SIHD. Fihn et al.
2.3 (pg 1933), Class IIa
Coronary angiography is reasonable in patients with suspected symptomatic SIHD who cannot undergo diagnostic stress testing, or have indeterminate or nondiagnostic stress tests, when there is a high likelihood that the findings will result in important changes to therapy. (Level of Evidence: C)

2014 ACC/AHA Multi-Societal Focused Update of the Guideline for the Diagnosis and Management of Patients with SIHD. Fihn et al.
2.3 (pg 1933), Class IIb
Coronary angiography might be considered in patients with stress test results of acceptable quality that do not suggest the presence of CAD when clinical suspicion of CAD remains high and there is a high likelihood that the findings will result in important changes to therapy. (Level of Evidence: C)

2.4 Other Cardiovascular Conditions in Patients Without Symptoms of Ischemia

Indication	Class of Rec. (COR)	Level of Evidence (LEVEL) Review	Reference
**Newly Diagnosed Heart Failure (Resting LV function previously assessed but No Prior CAD Evaluation)**			
51. Newly diagnosed HFpEF	**IIa**	**C**	**2013 ACCF/AHA Heart Failure Guideline. Yancy et al.**<br>**6.4 (pg e166)**<br>Noninvasive imaging to detect myocardial ischemia and viability is reasonable in patients presenting with de novo HF, who have known CAD and no angina, unless the patient is not eligible for revascularization of any kind.
	**IIa**	**C**	**2013 ACCF/AHA Heart Failure Guideline. Yancy et al.**<br>**6.5 (pg e167)**<br>When ischemia may be contributing to HF, coronary arteriography is reasonable for patients eligible for revascularization.
	**IIa**	**C**	**2014 ACC/AHA Multi-Societal Focused Update of the Guideline for the Diagnosis and Management of Patients with SIHD. Fihn et al.**<br>**2.3 (pg 1933)**<br>Coronary angiography is reasonable to define the extent and severity of coronary artery disease (CAD) in patients with suspected SIHD whose clinical characteristics and results of noninvasive testing (exclusive of stress testing) indicate a high likelihood of severe IHD and who are amenable to, and candidates for, coronary revascularization (7–12).
	**IIb**	**C**	**2014 ACC/AHA Multi-Societal Focused Update of the Guideline for the Diagnosis and Management of Patients with SIHD. Fihn et al.**<br>**2.3 (pg 1933)**<br>Coronary angiography might be considered in patients with stress test results of acceptable quality that do not suggest the presence of CAD when clinical suspicion of CAD remains high and there is a high likelihood that the findings will result in important changes to therapy.
52. Newly diagnosed HFrEF	**IIa**	**C**	**2013 ACCF/AHA Heart Failure Guideline. Yancy et al.**<br>**6.4 (pg e166)**<br>Noninvasive imaging to detect myocardial ischemia and viability is reasonable in patients presenting with de novo HF, who have known CAD and no angina, unless the patient is not eligible for revascularization of any kind.

	**IIa**	**C**	**2013 ACCF/AHA Heart Failure Guideline. Yancy et al.**<br>**6.5 (pg e167)**<br>When ischemia may be contributing to HF, coronary arteriography is reasonable for patients eligible for revascularization.
	**IIa**	**C**	**2014 ACC/AHA Multi-Societal Focused Update of the Guideline for the Diagnosis and Management of Patients with SIHD. Fihn et al.**<br>**2.3 (pg 1933)**<br>Coronary angiography is reasonable to define the extent and severity of coronary artery disease (CAD) in patients with suspected SIHD whose clinical characteristics and results of noninvasive testing (exclusive of stress testing) indicate a high likelihood of severe IHD and who are amenable to, and candidates for, coronary revascularization (7–12).
	**IIb**	**C**	**2014 ACC/AHA Multi-Societal Focused Update of the Guideline for the Diagnosis and Management of Patients with SIHD. Fihn et al.**<br>**2.3 (pg 1933)**<br>Coronary angiography might be considered in patients with stress test results of acceptable quality that do not suggest the presence of CAD when clinical suspicion of CAD remains high and there is a high likelihood that the findings will result in important changes to therapy.
53. Screening for transplant vasculopathy	**IIa**	**B**	**2011 ACCF/AHA/SCAI PCI Guideline. Levine et al.**<br>**5.4.2. IVUS (pg e73)**<br>IVUS and coronary angiography are reasonable 4 to 6 weeks and 1 year after cardiac transplantation to exclude donor CAD, detect rapidly progressive cardiac allograft vasculopathy, and provide prognostic information.
	**IIa**	**C**	**2014 ACC/AHA Multi-Societal Focused Update of the Guideline for the Diagnosis and Management of Patients with SIHD. Fihn et al.**<br>**2.3 (pg 1933)**<br>Coronary angiography is reasonable to define the extent and severity of coronary artery disease (CAD) in patients with suspected SIHD whose clinical characteristics and results of noninvasive testing (exclusive of stress testing) indicate a high likelihood of severe IHD and who are amenable to, and candidates for, coronary revascularization (7–12).
			**2014 ACC/AHA Multi-Societal Focused Update of the Guideline for the Diagnosis and Management of Patients with SIHD. Fihn et al.**

	IIb	C	**2.3 (pg 1933)**<br>Coronary angiography might be considered in patients with stress test results of acceptable quality that do not suggest the presence of CAD when clinical suspicion of CAD remains high and there is a high likelihood that the findings will result in important changes to therapy.
	I	C	**2015 Eligibility and Disqualification Recommendations for Competitive Athletes With Cardiovascular Abnormalities**<br>Thompson PD, Myerburg RJ, Levine BD, et al. Eligibility and disqualification recommendations for competitive athletes with cardiovascular abnormalities. Task Force 8: coronary artery disease: a scientific statement from the American Heart Association and American College of Cardiology. Circulation. 2015;132:e310–e314.<br>**Page e311**<br>Athletes with ASCAD should undergo maximal exercise testing to evaluate exercise tolerance, the presence of inducible ischemia, and the presence of exercise induced electrical instability. Testing should be performed on the subject's standard medical regimen, including b-adrenergic blocking medications.

Evaluation of Arrhythmias without Ischemic Equivalent (No Prior Cardiac Evaluation)			
54. Infrequent PVCs	IIa	C	**2014 EHRA/HRS/APHRS Expert Consensus on Ventricular Arrhythmias**<br>**Pedersen et al. 2014**<br>**Oct;11(10):e166-96.**<br>Infrequent ventricular ectopic beats, couplets, and triplets without other signs of an underlying SHD or an inherited arrhythmia syndrome should be considered as a normal variant in asymptomatic patients.
55. Frequent PVCs or nonsustained VT			
56. Paroxysmal supraventricular tachycardia			
57. New-onset atrial fibrillation /flutter			

58. Prior to initiation of antiarrhythmic therapy in patients with high global CAD risk		N	**2014 AHA/ACC/HRS Guideline for the Management of Patients With Atrial Fibrillation. January et al. J Am Coll Cardiol. 2014;64:e1–76.**<br>**Page e76.**<br>No Class level (from Appendix 4)<br>1.Exercise testing: To exclude ischemia before treatment of selected patients with a type IC antiarrhythmic drug
59. Exercise-induced VT	IIa<br><br>IIa<br><br>IIb	C<br><br>C<br><br>C	**2014 EHRA/HRS/APHRS Expert Consensus on Ventricular Arrhythmias Pedersen et al. 2014 Oct;11(10):e166-96.**<br>A test for myocardial ischaemia should be considered in all patients with VAs in whom the clinical presentation and/or the type of arrhythmia suggests the presence of coronary artery disease.<br><br>**2014 ACC/AHA Multi-Societal Focused Update of the Guideline for the Diagnosis and Management of Patients with SIHD. Fihn et al.**<br>**2.3 (pg 1933)**<br>Coronary angiography is reasonable to define the extent and severity of coronary artery disease (CAD) in patients with suspected SIHD whose clinical characteristics and results of noninvasive testing (exclusive of stress testing) indicate a high likelihood of severe IHD and who are amenable to, and candidates for, coronary revascularization (7–12).<br><br>**2014 ACC/AHA Multi-Societal Focused Update of the Guideline for the Diagnosis and Management of Patients with SIHD. Fihn et al.**<br>**2.3 (pg 1933)**<br>Coronary angiography might be considered in patients with stress test results of acceptable quality that do not suggest the presence of CAD when clinical suspicion of CAD remains high and there is a high likelihood that the findings will result in important changes to therapy.
60. Sustained VT	IIa<br><br>IIa	C<br><br>C	**2014 EHRA/HRS/APHRS Expert Consensus on Ventricular Arrhythmias Pedersen et al. Oct;11(10):e166-96.**<br>A test for myocardial ischaemia should be considered in all patients with VAs in whom the clinical presentation and/or the type of arrhythmia suggests the presence of coronary artery disease.<br><br>**2014 ACC/AHA Multi-Societal Focused Update of the Guideline for the Diagnosis and Management of Patients with SIHD. Fihn et al.**<br>**2.3 (pg 1933)**

			Coronary angiography is reasonable to define the extent and severity of coronary artery disease (CAD) in patients with suspected SIHD whose clinical characteristics and results of noninvasive testing (exclusive of stress testing) indicate a high likelihood of severe IHD and who are amenable to, and candidates for, coronary revascularization (7–12).
	**IIb**	**C**	**2014 ACC/AHA Multi-Societal Focused Update of the Guideline for the Diagnosis and Management of Patients with SIHD. Fihn et al. 2.3 (pg 1933)**<br>Coronary angiography might be considered in patients with stress test results of acceptable quality that do not suggest the presence of CAD when clinical suspicion of CAD remains high and there is a high likelihood that the findings will result in important changes to therapy.
61. Ventricular Fibrillation	**IIa**<br><br>**IIa**<br><br>**IIb**	**C**<br><br>**C**<br><br>**C**	**2014 EHRA/HRS/APHRS Expert Consensus on Ventricular Arrhythmias. Pedersen et al. Oct;11(10):e166-96.**<br>A test for myocardial ischaemia should be considered in all patients with VAs in whom the clinical presentation and/or the type of arrhythmia suggests the presence of coronary artery disease.<br><br>**2014 ACC/AHA Multi-Societal Focused Update of the Guideline for the Diagnosis and Management of Patients with SIHD. Fihn et al. 2.3 (pg 1933)**<br>Coronary angiography is reasonable to define the extent and severity of coronary artery disease (CAD) in patients with suspected SIHD whose clinical characteristics and results of noninvasive testing (exclusive of stress testing) indicate a high likelihood of severe IHD and who are amenable to, and candidates for, coronary revascularization (7–12).<br><br>**2014 ACC/AHA Multi-Societal Focused Update of the Guideline for the Diagnosis and Management of Patients with SIHD. Fihn et al. 2.3 (pg 1933)**<br>Coronary angiography might be considered in patients with stress test results of acceptable quality that do not suggest the presence of CAD when clinical suspicion of CAD remains high and there is a high likelihood that the findings will result in important changes to therapy.

**Syncope Without Ischemic Equivalent**			
62. Initial evaluation suggests CV abnormalities	**IIa**<br><br><br><br><br><br>**IIa**<br><br><br><br><br><br><br><br><br>**IIb**	**C**<br><br><br><br><br><br>**C**<br><br><br><br><br><br><br><br><br>**C**	**2014 EHRA/HRS/APHRS Expert Consensus on Ventricular Arrhythmias. Pedersen et al. 2014 Oct;11(10):e166-96.**<br>A test for myocardial ischaemia should be considered in all patients with VAs in whom the clinical presentation and/or the type of arrhythmia suggests the presence of coronary artery disease.<br><br>**2014 ACC/AHA Multi-Societal Focused Update of the Guideline for the Diagnosis and Management of Patients with SIHD. Fihn et al.**<br>**2.3 (pg 1933)**<br>Coronary angiography is reasonable to define the extent and severity of coronary artery disease (CAD) in patients with suspected SIHD whose clinical characteristics and results of noninvasive testing (exclusive of stress testing) indicate a high likelihood of severe IHD and who are amenable to, and candidates for, coronary revascularization (7–12).<br><br>**2014 ACC/AHA Multi-Societal Focused Update of the Guideline for the Diagnosis and Management of Patients with SIHD. Fihn et al.**<br>**2.3 (pg 1933)**<br>Coronary angiography might be considered in patients with stress test results of acceptable quality that do not suggest the presence of CAD when clinical suspicion of CAD remains high and there is a high likelihood that the findings will result in important changes to therapy.
63. Initial evaluation suggests other etiology			
Cardio-oncology			
64. Prior chest radiation, no symptoms, >5 y ago			

References:

2014 ACC/AHA Multi-Societal Focused Update of the Guideline for the Diagnosis and Management of Patients with SIHD. Fihn et al.
2.3 (pg 1933), Class IIa
Coronary angiography is reasonable to define the extent and severity of coronary artery disease (CAD) in patients with suspected SIHD whose clinical characteristics and results of noninvasive testing (exclusive of stress testing) indicate a high likelihood of severe IHD and who are amenable to, and candidates for, coronary revascularization (7–12). (Level of Evidence: C)

2014 ACC/AHA Multi-Societal Focused Update of the Guideline for the Diagnosis and Management of Patients with SIHD. Fihn et al.
2.3 (pg 1933), Class IIa
Coronary angiography is reasonable in patients with suspected symptomatic SIHD who cannot undergo diagnostic stress testing, or have indeterminate or nondiagnostic stress tests, when there is a high likelihood that the findings will result in important changes to therapy. (Level of Evidence: C)

2014 ACC/AHA Multi-Societal Focused Update of the Guideline for the Diagnosis and Management of Patients with SIHD. Fihn et al.
2.3 (pg 1933), Class IIb
Coronary angiography might be considered in patients with stress test results of acceptable quality that do not suggest the presence of CAD when clinical suspicion of CAD remains high and there is a high likelihood that the findings will result in important changes to therapy. (Level of Evidence: C)
