# Supplementary material for: ACC/AHA/ASE/ASNC/ASPC/HFSA/HRS/SCAI/SCCT/SCMR/STS 2023 Multimodality Appropriate Use Criteria for the Detection and Risk Assessment of Chronic Coronary Disease
Source: J Cardiovasc Magn Reson. 2023 Oct 19;25:58. doi: 10.1186/s12968-023-00958-5 (PMC10585920; doi:10.1186/s12968-023-00958-5)
Supplement: Supplementary file 2 — Additional file 2. Relationships with Industry and Other Entities (Comprehensive). [file 12968_2023_958_MOESM2_ESM.pdf]

2023 ACC/AHA/ASE/ASNC/ASPC/HFSA/HRS/SCAI/SCCT/SCMR/STS Multimodality Appropriate Use Criteria for the Detection and Risk Assessment of Chronic Coronary Disease: Members of the Writing Group, Rating Panel, External Reviewers, and Solution Set Oversight Committee—Relationships with Industry and Other Entities (Comprehensive)

Relationships With Industry (RWI) And Other Entities

The ACC and its partnering organizations rigorously avoid any actual, perceived, or potential conflicts of interest that might arise as a result of an outside relationship or personal interest of a member of the rating panel. Specifically, panelists are asked to provide disclosure statements of all relationships that might be perceived as real or potential conflicts of interest. These statements were discussed with all members of the rating panel at the virtual rating panel meeting and updated and reviewed as necessary. The following is a full list of disclosures by all participants, including relationships not pertinent to this document.

Participant	Employment	Representing	Consultant	Speakers Bureau	Ownership/ Partnership/ Principal	Personal Research	Institutional, Organizational, or Other Financial Benefit	Expert Witness
				**Writing Group**				
David E. Winchester, *Co-Chair*	University of Florida, Division of Cardiology—Professor of Medicine and Radiology	ACC	None	None	None	None	None	None
David J. Maron, *Co-Chair*	Stanford University School of Medicine—Professor of Medicine, Cardiovascular, Director, Preventive Cardiology	ACC	None	None	None	• NIH/NHLBI (ISCHEMIA)	• Ablative Solutions*<br>• Medscape (Member, Advisory Group)	None
Ron Blankstein	Brigham and Women's Hospital—Associate Director, Cardiovascular Imaging Program, Professor of Medicine, Harvard Medical School	SCCT	• Amgen, Inc<br>• Caristo Diagnostics<br>•Novartis†	None	None	• Amgen, Inc*<br>•Novartis•	• SCCT, (Officer)	None
Ian C. Chang	Mayo Clinic—Assistant Professor of Medicine,	ACC	None	None	None	None	None	None

Participant	Employment	Representing	Consultant	Speakers Bureau	Ownership/ Partnership/ Principal	Personal Research	Institutional, Organizational, or Other Financial Benefit	Expert Witness
	and Georgetown University—Assistant Professor of Medicine							
Ajay J. Kirtane	Columbia University Medical Center—Professor of Medicine, Chief Academic Office, Director NYP/Columbia Cardiac Cath	SCAI	• IMDS	None	None	• Abbott Vascular† • Amgen, Inc† • Boston Scientific† • Cardiovascular Systems, Inc† • CSI† • Medtronic† •Opsens • Philips/ Spectranetics† • ReCor Medical† •Regeneron •Zoll	None	None
Raymond Y. Kwong	Brigham and Women's Hospital—Director of Cardiac Magnetic Resonance Imaging	SCMR	None	None	None	• Alnylam, Inc† • MyoKardia, Inc.†	• SCMR, (Officer)*	None
Patricia A. Pellikka	Mayo Clinic College of Medicine—Professor of Medicine	ASE	• Ultromics • UpToDate†	None	None	• Bracco* • Edwards Lifesciences • GE Healthcare* • Lantheus Medical Imaging* • OxThera* • Ultromics	• ASE Foundation* • NHLBI*	None
Jordan M. Prutkin	Washington Medical	HRS	None	None	None	None	• UpToDate†	None

Participant	Employment	Representing	Consultant	Speakers Bureau	Ownership/ Partnership/ Principal	Personal Research	Institutional, Organizational, or Other Financial Benefit	Expert Witness
	University—Professor of Medicine							
Raymond Russell	Alpert Medical School of Brown University—Professor of Medicine	ASNC	None	None	• Dicerna† • Terns Pharmaceutical†	None	• Dicerna† • Terns Pharmaceutical (Officer)†	None
Alexander T. S. Sandhu	Stanford University School of Medicine—Instructor of Medicine	ACC	None	None	None	None	None	None
**Rating Panel**								
W. Patricia Bandettini	National Institutes of Health—Medical Officer, Heart Failure & Arrhythmias Branch	SCMR	None	None	None	None	None	None
Dennis A. Calnon	Ohio Health Heart and Vascular Physicians—Riverside Methodist Hospital, Cardiac Imaging, Director	ASNC	None	None	None	None	• ASNC, (Officer)	None
Manuel D. Cerqueira	Cleveland Clinic Foundation—Chairman, Department of Molecular and Functional Imaging	ACC Imaging Council	• Astellas Pharma†	• Astellas Pharma†	None	None	None	None
Larry S. Dean	Medicine Regional Heart Center University of Washington School of Medicine—Professor of Medicine and Surgery, Director	ACC	• ABIM • Teleflex	None	• Emageon	• Edwards Life Sciences†	•University of Washington Medicine (Member) †	None

Participant	Employment	Representing	Consultant	Speakers Bureau	Ownership/ Partnership/ Principal	Personal Research	Institutional, Organizational, or Other Financial Benefit	Expert Witness
Milind Y. Desai	Cleveland Clinic Foundation, Heart and Vascular Institute—Professor of Medicine	ACC	• Bristol Myers Squibb† • Caristo Diagnostics • Medtronic	None	None	None	None	None
Howard J. Eisen	Pennsylvania State Heart and Vascular Institute—Medical Director, Advanced Heart Failure, Cardiac Transplant Programs	ACC	None	None	None	None	None	None
Stephen E. Fremes	Sunnybrook Health Sciences Centre, Division of Cardiac and Vascular Surgery—Professor, Department of Surgery	ACC	None	None	None	None	• Bayer (COMPASS)‡ • Bayer (Galileo)‡ • Boston Scientific (NeoAcurate II Study) ‡ • Edwards (The Multidisciplina-ry, Mutltimoda-lity but Minima-list (3M) Approach to Transfemoral Transcatheter Aortic Valve Replacement)‡ • HLT, Inc (Radiant study)‡ • Medtronic (Medtronic TAVR Low Risk)‡ • Medtronic (SURTAVI)‡ • Medtronic	None

Participant	Employment	Representing	Consultant	Speakers Bureau	Ownership/ Partnership/ Principal	Personal Research	Institutional, Organizational, or Other Financial Benefit	Expert Witness
							(Evolut-R FORWARD)‡<br>• Bernard Goldman Chair in Cardiovas-cular Surgery†	
Mario F. L. Gaudino	Weill Cornell Medical College—Stephen and Suzanne Weiss Professor in Cardiothoracic Surgery, Professor of Cardiothoracic Surgery	STS	None	None	None	• Canadian Institutes of Health and Research*<br>• NIH*	None	None
Linda D. Gillam	Morristown Medical Center, Department of Cardiovascular Medicine—Chair	ASE	• Edwards Lifesciences†<br>• Egnite<br>• Medtronic†<br>• Philips†	None	None	• Edwards Lifesciences†<br>• Medtronic†	• Circulation Imaging (Officer)	None
Nicole L. Lohr	Medical College of Wisconsin, Cardiovascular Medicine—Professor of Medicine	AHA	None	None	None	• Amgen (Galactic, Heart Failure)‡	None	None
Joseph E. Marine	Johns Hopkins University School of Medicine, Cardiovascular Medicine—Professor of Medicine	HRS	None	None	None	None	None	None
Khurram Nasir	Houston Methodist DeBakey Cardiology Associates, Preventive Cardiology—Professor of Cardiology	ASPC	• Amgen, Inc†<br>• Esperion<br>• Novartis<br>• Novo Nordisk†	• Amgen, Inc.	None	None	None	None

Participant	Employment	Representing	Consultant	Speakers Bureau	Ownership/ Partnership/ Principal	Personal Research	Institutional, Organizational, or Other Financial Benefit	Expert Witness
Leslee J. Shaw	Icahn School of Medicine at Mount Sinai Hospital Mount Sinai—Director, Blavatnik Family Women's Health Research Institute, Professor of Medicine	SCCT	None	None	None	None	None	None
Jacqueline E. Tamis-Holland	Icahn School of Medicine at Mount Sinai Hospital Mount Sinai—Director, Women's Heart NY, Assistant Professor of Medicine, Director, Interventional Cardiology Fellowship	SCAI	• EBIX<br>• Gaffney Events Educational Trust	None	None	None	• AHA (Committee Member)*<br>• Pfizer Inc.	None
L. Samuel Wann	Cardiovascular Disease Consultant	ACC	None	None	None	None	None	None
John B. Wong	Tufts University School of Medicine—Professor of Medicine	ACC	• Informed Medical Decisions Foundation: Healthwise<br>• Annals of Internal Medicine (American College of Physicians)†	None	None	• Patient-Centered Outcomes Research Institute (PI)†	• United States Preventive Services Task Force (Member)	None
**Reviewers**								
Niti R. Aggarwal	Mayo Clinic— Assistant Professor of Medicine	Lead SSOC Reviewer	None	None	None	None	None	None
Daniel S. Berman	Cedars-Sinai Medical Center, Department of Imaging—Director,	ACC Imaging Council & SCCT	• Cedars Sinai Medical Center – Software	None	None	None	None	None

Participant	Employment	Representing	Consultant	Speakers Bureau	Ownership/ Partnership/ Principal	Personal Research	Institutional, Organizational, or Other Financial Benefit	Expert Witness
	Cardiac Imaging		Royalties†<br>• General Electronic					
Matthew J. Budoff	Los Angeles Biomedical Research Institute—Program Director, Division of Cardiology	SCCT	• Esperion†	• Amarin†<br>• Amgen, Inc†<br>• AstraZeneca Pharmaceuticals†<br>• Boehringer Ingelheim Pharmaceuticals†<br>• Novo Nordisk†	None	None	None	None
Andrew J. Einstein	Columbia University Irving Medical Center, Department of Medicine—Associate Professor of Medicine in Radiology	ASNC	• Actinia	None	None	• Canon Medical Systems†<br>• GE Healthcare†<br>• Roche Medical Systems†<br>• W. L. Gore†<br>• Novo Nordisk†	• ASNC (Officer)*<br>• JACC Imaging (Officer)*<br>• Journal of Nuclear Cardiology (Officer)*	None
Victor A. Ferrari	Hospital of the University of Pennsylvania—Professor of Medicine; Associate Director, Cardiovascular Imaging	SCMR	None	None	None	None	• Journal of Cardiovascular Magnetic Resonance (Officer)*	None
Theodore J. Kolias	University of Michigan Cardiovascular Center—Associate Professor of Medicine	ASE	None	None	None	None	None	None
Jonathon Leipsic	University of British Columbia, Department of Radiology—Professor of Radiology and Cardiology	SCCT	• CIRCL†<br>• MVRX†	• GE Healthcare<br>• Philips	• CIRCL CVI†<br>• Heartflow†<br>.	• Heartflow Inc†	• Abbott<br>• Boston Scientific<br>• Edwards<br>• Medtronic	None

Participant	Employment	Representing	Consultant	Speakers Bureau	Ownership/ Partnership/ Principal	Personal Research	Institutional, Organizational, or Other Financial Benefit	Expert Witness
Brian Olshansky	University of Iowa Carver College, Division of Electrophysiology—Emeritus Professor of Medicine	HRS	None	None	None	None	• Astra Zeneca (DSMB)	None
Harmony R. Reynolds	NYU Grossman School of Medicine, Department of Medicine—Associate Professor of Medicine	AHA	None	None	None	• Abbott Vascular* • Philips* • Siemens*	None	None
Peter P. Toth	University of Illinois College of Medicine, Division of Cardiology—Adjunct Professor of Medicine	ASPC	• Amarin • Kowa†	• Amgen, Inc† • Esperion • Novo-Nordisk†	None	None	None	None
Howard S. Weintraub	NYU Grossman School of Medicine, Department of Medicine—Clinical Professor of Medicine	ASPC	• Amgen, Inc. • Novartis	None	None	• Akcea† • Amarin • Amgen, Inc†	None	None
David H. Wiener	Jefferson Medical College, Jefferson Heart Institute—Professor of Medicine	ASE	None	None	None	None	None	None
**Solution Set Oversight Committee**								
RWI and disclosure statements for members of the SSOC can be found here: https://www.acc.org/guidelines/about-guidelines-and-clinical-documents/guidelines-and-documents-task-forces								

This table represents *comprehensive* relationships of participants with industry and other entities that were reported at the time this document was under development. The table does not necessarily reflect relationships with industry at the time of publication.

A person is deemed to have a *significant* interest in a business if the interest represents ownership of ≥5% of the voting stock or share of the business entity, or ownership of ≥$5,000 of the fair market value of the business entity; or if funds received by the person from the business entity exceed 5% of the person's gross income for the previous year. Relationships in this table with no symbol are considered *modest* (less than significant under the preceding definition). Relationships that exist with *no financial benefit* are also included for the purpose of transparency. Please refer to http://www.acc.org/guidelines/about-guidelines-and-clinical-documents/relationships-with-industry-policy for definitions of disclosure categories or additional information about the ACC Disclosure Policy for Writing Committees.

* No financial benefit
† Significant relationship
‡ Clinical Trial Enroller

ACC = American College of Cardiology; AHA = American Heart Association; ASE = American Society of Echocardiography; ASNC = American Society of Nuclear Cardiology; ASPC = American Society of Preventive Cardiology; AUC = Appropriate Use Criteria; DSMB = Data Safety Monitoring Board; HRS = Heart Rhythm Society; NIH = National Institutes of Health; NHLBI = National Heart, Lung, and Blood Institute; SCAI = Society for Cardiovascular Angiography and Interventions; SCCT = Society of Cardiovascular Computed Tomography; SCMR = Society for Cardiovascular Magnetic Resonance; SSOC = Solution Set Oversight Committee.
